# A Study of Word Complexity Under Conditions of Non-experimental, Natural Overt Speech Production Using ECoG

**DOI:** 10.3389/fnhum.2021.711886

**Published:** 2022-02-04

**Authors:** Olga Glanz, Marina Hader, Andreas Schulze-Bonhage, Peter Auer, Tonio Ball

**Affiliations:** ^1^GRK 1624 “Frequency Effects in Language,” University of Freiburg, Freiburg, Germany; ^2^Department of German Linguistics, University of Freiburg, Freiburg, Germany; ^3^The Hermann Paul School of Linguistics, University of Freiburg, Freiburg, Germany; ^4^BrainLinks-BrainTools, University of Freiburg, Freiburg, Germany; ^5^Neurobiology and Biophysics, Faculty of Biology, University of Freiburg, Freiburg, Germany; ^6^Translational Neurotechnology Lab, Department of Neurosurgery, Faculty of Medicine, Medical Center—University of Freiburg, University of Freiburg, Freiburg, Germany; ^7^Department of Neurosurgery, Faculty of Medicine, Epilepsy Center, Medical Center—University of Freiburg, University of Freiburg, Freiburg, Germany; ^8^Bernstein Center Freiburg, University of Freiburg, Freiburg, Germany

**Keywords:** natural behavior, spontaneous speech production, ECoG, word complexity, articulation

## Abstract

The linguistic complexity of words has largely been studied on the behavioral level and in experimental settings. Only little is known about the neural processes underlying it in uninstructed, spontaneous conversations. We built up a multimodal neurolinguistic corpus composed of synchronized audio, video, and electrocorticographic (ECoG) recordings from the fronto-temporo-parietal cortex to address this phenomenon based on uninstructed, spontaneous speech production. We performed extensive linguistic annotations of the language material and calculated word complexity using several numeric parameters. We orthogonalized the parameters with the help of a linear regression model. Then, we correlated the spectral components of neural activity with the individual linguistic parameters and with the residuals of the linear regression model, and compared the results. The proportional relation between the number of consonants and vowels, which was the most informative parameter with regard to the neural representation of word complexity, showed effects in two areas: the frontal one was at the junction of the premotor cortex, the prefrontal cortex, and Brodmann area 44. The postcentral one lay directly above the lateral sulcus and comprised the ventral central sulcus, the parietal operculum and the adjacent inferior parietal cortex. Beyond the physiological findings summarized here, our methods may be useful for those interested in ways of studying neural effects related to natural language production and in surmounting the intrinsic problem of collinearity between multiple features of spontaneously spoken material.

## Introduction

### What Is “Word Complexity”?

There is a body of psycho- and neurolinguistic research dedicated to word complexity, which has been motivated by clinical interest in improving the speakers’ verbal capacities after neurological impairments affecting speech. Previous research has identified such relevant factors as word length, the position of phonemes within a word, particularly the association of stronger impairments in phoneme production at word-initial positions, and the occurrence of consonant clusters as important predictors of articulatory errors (reviewed in Ziegler and Aichert, 2015). This research, however, has largely been conducted in patients with neurological disorders, and little is known about how the linguistically unimpaired human brain processes words of different complexity. In the present study, we have addressed the modulation of neural activity by word complexity metrics in freely speaking linguistically unimpaired individuals.

According to Miestamo (2006a,b), linguistic complexity can be defined in two general ways: the “absolute” and the “relative.” The absolute approach aims at objective, theory-based descriptions which account for the number of structural units and rules involved in a system. From this perspective, the more rules and parts are involved, the more complex a language or language unit is. The relative approach, on the contrary, is not theory- but usage-based. It defines linguistic complexity based on how difficult a particular language or a linguistic unit is for a language user, regardless of the structural properties of the linguistic material.

Since linguistic complexity can have multiple facets, previous psycholinguistic research on word complexity has considered numerous linguistic parameters simultaneously (Ziegler and Aichert, 2015). We have also used several parameters to operationalize the complexity of individual content words so as not to overlook potentially relevant aspects of the linguistic material. The measures we have implemented are (i) the number of spoken syllables in a word (NoS), (ii) the consonant-to-vowel ratio (CVR), calculated by dividing the number of consonants by the number of vowels in the spoken word, (iii) an “ease-of-articulation” (EoA) index, calculated according to the model by Ziegler and Aichert (2015), and (iv) the lemma frequency of the words (FRQ). It is intuitive to assume that the more syllables a word has, the more complex it is. CVR was chosen because consonants can be considered more complex than vowels: Shankweiler and Harris (1966), for instance, found that the production of vowels in patients with aphasia was less affected than the production of consonants, lending support to the idea that vowels are easier to articulate. Also, consonants are mastered more slowly than vowels, and advances in L1 consonant mastery are the best predictors of lexical advance within the first years of life (Vihman, 2014). These observations support the idea that consonants contribute to more effort in speech production. We chose FRQ as a measure of word complexity since frequent words are associated with fewer processing errors and faster reaction times, indicating their greater ease for the language user (Bybee, 2010).

The EoA index by Ziegler and Aichert (2015) presents a complex measure including several aspects of articulatory phonology. It arose from these researchers’ investigations on what aspects of words are predictive of articulatory errors in patients suffering from apraxia of speech. Aichert and Ziegler (2004) and Ziegler and Aichert (2015) identified the metric pattern, the occurrence of complex constrictions, as well as the number of articulatory gestures involved in word production (i.e., discrete actions of the lips, the tongue, the velum and the glottis) as important error predictors and included them in an EoA model, which they trained and validated. Thus, a special feature of the EoA index is that, unlike the rest of the parameters we have analyzed, it accounts for multiple parameters related to linguistic complexity.

### “The Freiburg Neurolinguistic Corpus”

This study was conducted using spontaneously spoken data together with simultaneous electrocorticographic (ECoG) recordings. We created a multimodal neurolinguistic corpus “The Freiburg Neurolinguistic Corpus” (FNLC) consisting of simultaneous video, audio, and neural recordings obtained during overt, spontaneous, uninstructed speech production of epilepsy patients while they were engaged in face-to-face communication with visitors or medical personnel (see section “Materials and Methods”).

### The Neurolinguistic Hypothesis in the Light of Previous Psycho- and Neurolinguistic Findings

We were looking for neural indications of effort associated with the production of words of varying complexity. Based on previous evidence, we assumed that linguistically complex words would be more difficult to process and that they would hence be associated with neural markers reflecting increased effort during speech production. Previous research has shown that high-frequency activity in the gamma range > 35 Hz is a spatially, temporally and functionally specific index of event-related cortical activation (Pfurtscheller and Da Silva, 1999, Crone et al., 2011) and that gamma activity is proportionally modulated by the difficulty of the task (Senkowski and Herrmann, 2002). We thus expected that high CVR, high NoS, low EoA, and low FRQ, which can be seen as linguistically complex for the reasons mentioned above, would be associated with increased spectral magnitude values in gamma frequencies. If one correlates the aforementioned parameters with gamma activity, one would thus expect positive correlations with CVR and NoS but negative correlations with EoA and FRQ in language-relevant cortical areas (Hickok, 2012).

A characteristic feature of gamma-range speech-related cortical activation in our previous studies was that it presented a homogeneous, broad-banded response extending over multiple frequencies, typically strongest between 70 and 150 Hz (e.g., Derix et al., 2014; Glanz et al., 2018). We were therefore predominantly anticipating effects in high gamma frequencies.

## Materials and Methods

### Subjects

**SCHEME 1:**
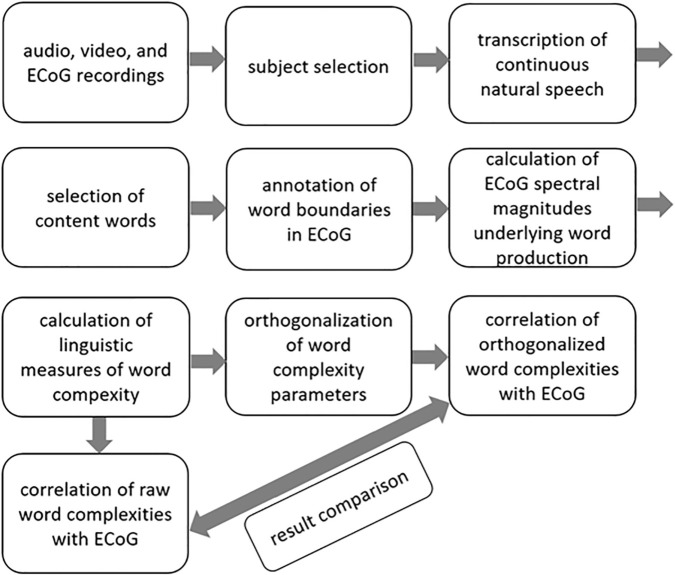
A brief overview of the methods.

Data from five native speakers of German (Table 1) were analyzed. We transcribed the subjects’ continuous speech production (Selting et al., 2009) and extracted simple clauses from the spoken data. Within the borders of simple clauses, we identified individual content words and annotated them with regard to syllable structure, lexical frequency, and several prosodic features. We then manually identified the simple clauses and the content words in the neural data and marked their beginnings and ends to be able to align the linguistic analyses with the ECoG recordings. Detailed information on what linguistic decisions have been made and what theoretical considerations they are based on is available elsewhere (Neuromedical AI Lab, 2019). A brief overview of the methods is provided in Scheme 1.

**TABLE 1:** Subject details.

s.	*S*1	*S*2	*S*3	*S*4	*S*5
Age	22	42	29	49	41
Sex	M	M	F	F	F
Handedness	R	R	R	R*	R
Speech lateralization	L	L	L	B	L
Location of the 8 × 8 electrode grid	L FTP	L FTP	L FTP	L FTP	L FTP
Seizure onset zone	IH, PF, PM	IP, PM, TC, TB	IH, IP, PO, TC, TB	PF, PM	IH, PM

s, subject; M, male; F, female; R, right; B, bilateral; L, left; R*, right-handed converted from left; FTP, fronto-temporo-parietal; FP, fronto-polar; OP, occipito-parietal; IH, interhemispheric; IP, inferior parietal cortex; PO, parietal operculum; PF, prefrontal cortex; PM, premotor cortex; TC, temporal cortex; TB, temporo-basal cortex.

A major criterion for subject selection was that the seizure onset zone, identified in continuous ECoG recordings during pre-neurosurgical evaluation of epilepsy, did not overlay potentially speech-relevant cortical areas. These were identified by electrocortical stimulation mapping (ESM) as areas implicated in speech and mouth motor functions. All subjects gave written informed consent that all audio, video, and neural data obtained in the course of pre-neurosurgical diagnostics would be made available for research, and the Ethics Committee of the University Medical Center Freiburg approved the protocol for subject recruitment. Both ECoG and ESM data were gathered by trained medical personnel at the University Medical Center Freiburg. The locations and numbers of electrodes were defined by the subjects’ clinical needs. The study was performed retrospectively upon completion of diagnostics, and it did not interfere with the pre-neurosurgical procedures.

**FIGURE 1:**
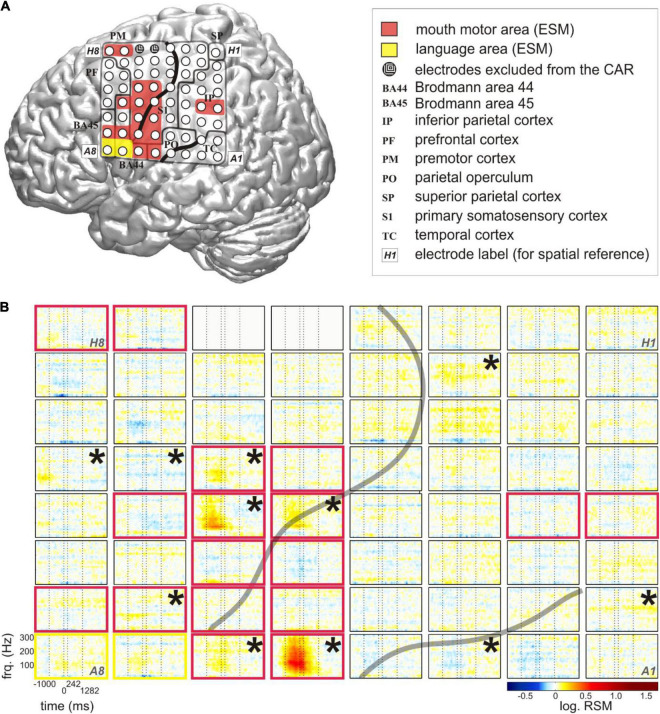
Typical relative spectral magnitude (RSM) responses underlying the production of content words in relation to the individual cortical architecture (S5). **(A)** Anatomical location of the electrode grid projected onto the standard brain surface from spm5 based on the Montreal Neurological Institute (MNI) coordinates of the individual electrodes. Rows of the electrode grid are labeled by letters A to H, and the columns are labeled by numbers 1–8. A1 to H8: labels of the individual electrodes of the grid, included for ease of spatial reference. Conventions for structural and functional anatomy: see legend. Gray solid lines indicate the borders of anatomical areas. Black solid lines indicate individual positions of the central and lateral sulci. Color coding of the overlays highlights potentially speech-relevant areas identified with the help of ESM. **(B)** The responses at individual electrodes of the 8 × 8 grid are shown for frequencies up to 300 Hz. The transparent gray lines indicate the positions of the individual central and lateral sulci. Electrodes in light gray (H5, H6) lay in the seizure onset zone. They were excluded from the common average reference (CAR) and from all analyses. Vertical dashed lines at each of the 64 electrode panels indicate, from left to right, the average speech start, word start, word end, and speech end. The black stars in the upper right corners of the electrode panels mark the electrodes with significant RSM increases in gamma frequencies during the depicted time window (Wilcoxon sign test, FDR-corrected at *q* < 0.05). Color coding of the electrode panels corresponds to the ESM-identified speech-relevant areas in **(A)**.

ECoG recordings were acquired with platinum/stainless-steel electrodes (4-mm diameter, 10-mm center-to-center inter-electrode distance) at the Epilepsy Center, University Medical Center Freiburg. An EEG system with a sampling rate of 1,024 Hz was used with a high-pass filter using a cut-off frequency of 0.032 Hz and a low-pass, anti-aliasing filter at 379 Hz. The subjects were monitored with around-the-clock audio and video recordings synchronized with the ECoG signal. The video recordings had a resolution of 640 × 480 pixels and a sampling rate of 25 Hz. All patients had 8 × 8 electrode grids in comparable locations of the left fronto-temporo-parietal cortex (Figure 1A).

### Assignment of Electrodes to Anatomical and Functional Areas

The assignment of electrodes to anatomical areas was performed using the same hierarchical probabilistic method of anatomical assignment as in our previous studies (e.g., Derix et al., 2012, 2014; Ruescher et al., 2013). We used ESM information to define potentially language-relevant cortical areas including those engaged in movements of mouth motor effectors such as the tongue, the lips, the cheeks or those implicated in cognitive language functions. Whether the word-related effects were located within or adjacent to these potentially language-relevant areas was important for the functional interpretation of the effects.

### Pre-selection of the Spoken Material

Upon visual screening of the entire around-the-clock data, we pre-selected recordings in which the subjects were awake, alert, and participating in face-to-face conversations with visitors, hospital ward neighbors, or medical personnel. The numbers of hours of recordings we selected per subject depended on the overall duration of their hospital stay, on how much they spoke on average, and on the intelligibility of the language material. Acoustically and mechanically undistorted speech production epochs were analyzed. To avoid erroneous transcriptions, we discarded recordings obtained when the subjects were eating or drinking, speaking quietly, speaking in the presence of loud acoustic background noise as well as during long periods of overlapping talk.

### Transcription of the Spoken Material and Word Extraction

Trained linguists performed transcriptions of the patients’ speech using the freeware PRAAT (Boersma and Van Heuven, 2001) in accordance with the “basic” transcript of the GAT-2 conventions (Selting et al., 2009). Then, we identified simple clauses within the borders of the acquired intonation phrases, which was part of the acquisition of the FNLC (Iljina et al., 2017; Diekmann, 2019; Glanz, 2021; Supplementary Appendices 1.1, 1.2).

We extracted content words from the clauses. Each word in each simple clause was subject to a PoS analysis, conducted according to the Stuttgart-Tübingen-Tag-Set (STTS) conventions for the German language (Schiller et al., 1995). We modified the tag set in order to differentiate between adverbs and homophone particles, which can only be distinguished from each other in German with the help of the surrounding semantic and phonological context. The PoS annotation was conducted by hand, since automated taggers are not sensitive to such context information (Neuromedical AI Lab, 2019). Unlike STTS, we also differentiated between homophone full verbs, copula verbs, and auxiliaries. The spelling of the tags was checked with the help of a custom-made MATLAB-based program to eliminate errors due to manual annotation. This program also checked if the number of words in a clause was the same as the number of PoS tags for the respective clause, it identified impossible or statistically unlikely combinations of linguistic tags in the same clause (e.g., two or more non-finite verbs in a row etc.), and displayed them to the investigator for validation. There is evidence that neural activity can differ between content and function words (Münte et al., 2001; Diaz and McCarthy, 2009). We used content words only to have homogeneous samples. All words of the PoS categories “full verb” (FV), “normal noun” (NN), and “adverb” (ADV) were extracted from the clauses and ordered chronologically within the respective PoS category. The ADV category consisted of adverbs (ADV in STTS), adverbial adjectives (i.e., adjectives used as adverbs, ADJD in STTS), interrogative or relative adverbs (PWAV), and pronominal adverbs (PAV). To avoid the dominance of repeated words, we discarded all repetitions of the same word in the word list analyzed within each subject and used the word only in its first occurrence. Since the EoA index (Ziegler and Aichert, 2015) can only be calculated for words whose phonological length does not exceed three syllables, we limited the selection of words accordingly to meet this criterion.

### Lemmatization and Extraction of Lemma Frequencies

We used lemma frequency information. Word lemma forms were extracted with the help of the lemmatization tool of the online platform WEBLICHT (Hinrichs et al., 2010), designed for the automated generation of corpora. We obtained lemma frequency information from the Forschungs- und Lehrkorpus Gesprochenes Deutsch or FOLK (Engl. “Research and Teaching Corpus of Spoken German”), developed by the Institut für Deutsche Sprache (Engl. “Institute for the German Language,” FOLK, 2012). We took the PoS information into account to avoid erroneous frequency assignments from homographs of a different PoS category. For instance, the lemma “sein” (Engl. “to be”) was listed in FOLK in finite and non-finite auxiliary verb forms but also as an attributive possessive pronoun (Engl. “his”), and we used the sum of the frequencies of the verb forms only, whenever “sein” was used as a verb.

### Annotation of the Speech-Accompanying Electrocorticographic Data

**FIGURE 2:**
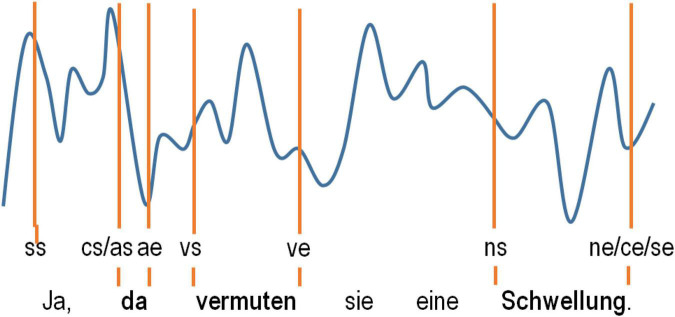
A schematic representation of how the tagging of the words selected in the ECoG data was carried out. The tags used denote the speech start (ss) and speech end (se) and the start and end of the words selected (as/ae for adverbs, ns/ne for nouns and vs/ve for verbs).

The next step of data acquisition was identification of the pre-selected content words and their embedding language sequences in the neural data with the help of the software Coherence EEG/PSG System (Deltamed: Paris, France), which was also used to record the data. This software allows for simultaneous visualization of raw ECoG potentials at each of the recorded electrodes together with synchronized video and audio materials. Since precise automated tagging of the neurolinguistic data using Coherence was not possible for technical reasons, we opted for manual tagging of the speech-accompanying neural data. The tagging of each word was performed manually using PoS-specific markers for word starts and ends based on aural inspection of the acoustic information in the video recordings frame by frame. We also tagged the starts and ends of the embedding clause boundaries (“cs” and “ce” for the start and end, respectively). We tagged the starts and ends of the embedding speech production epoch (“ss” and “se”) in which the words occurred to be able to account for the position of the words and clauses within these language units. Clauses were tagged whenever they lay within the borders of a single intonation phrase (see Supplementary Appendix 1.2.1 for details on our linguistic definition of a “clause”). Since psycholinguistic research has shown that humans perceive pauses ≥ 200 ms as actual “pauses” in a conversation (Walker and Trimboli, 1982), we selected clauses with such pauses for the sake of homogeneity of our linguistic material. A “speech production epoch” was defined by the absence of pauses ≥ 200 ms. The tagging procedure is illustrated in Figure 2.

The annotators were instructed to make sure that the tags for clause boundaries were set as precisely as possible, whenever multiple tags had to be set simultaneously, and validation of each tag by at least two other people was performed to control for their temporal precision. Due to the manual nature of our annotations, however, the word start tag could slightly precede the tag for speech/clause start tag within the same speech production epoch, and the word/clause end tags could follow the corresponding speech end tag by several ms. Automated *post hoc* correction using custom-made Matlab-based software was applied to account for such imprecisions (Supplementary Appendix 1.5).

### Spectral Analysis of the Electrocorticographic Data

ECoG data were re-referenced to a common average reference (CAR). All electrodes with artifacts caused by impaired connections to the amplifier and those lying in the seizure onset zone were excluded from the CAR and removed from subsequent analyses (gray-colored circles in Figure 1A). The total duration of the time window analyzed was 2 s before and 3 s after word onset. The frequency range analyzed was up to 300 Hz. The data cut out relative to word start within the time-frequency window selected will be referred to as “trials.” We computed word-onset-related, time-resolved spectral magnitude changes for all words of each subject using a Fast Fourier transformation. 200-ms sliding windows and 20-ms time steps were used for calculation of the absolute spectral magnitudes (ASM). This yielded a frequency resolution of ca. 5 Hz and a time resolution of ca. 200 ms. (We also probed alternative settings with 500-ms sliding windows and 50-ms time steps. They proved consistently worse in statistical analyses, which is likely due to inferior temporal resolution. For this reason, we will refrain from reporting on them in the following.) We applied a multitaper method (Percival, 2000) with 5 Slepian tapers to diminish the cutting artifacts at the edges of the temporal sequences analyzed.

### Baseline Correction

The ASM in each trial was baseline-corrected. The baseline was generated by averaging the frequency-resolved ASM in the respective trial over the first 200 ms of the time window analyzed, corresponding to [−2 −1.8] s relative to the start of each word. The relative spectral magnitudes (RSM) obtained for each word, ordered chronologically within the respective PoS in the given subject, were concatenated for all three PoS categories. The ASM values for each trial were divided, in a time- and frequency-resolved manner, by the resulting baseline. The RSM were transformed to a natural logarithmic scale using the log function implemented in MATLAB, trial-averaged and visualized using custom-made MATLAB-based software (Figure 1B).

### Statistical Analyses of the Electrocorticographic Data

We compared the RSM values in all time-frequency bins for all trials at each electrode against the value of “1.” The *p*-values obtained were corrected for multiple comparisons for the number of time bins, frequency bins, and the number of tested grid electrodes (Wilcoxon sign test, FDR-corrected at *q* < 0.05, Figure 1B).

### Acquisition of Linguistic Parameters Depending on Word-, Clause-, and Speech-Epoch Duration

The electrode grids in all subjects covered extensive portions of the motor cortex (e.g., Figure 1A), which contributes to low-level processes related to the execution of motor actions (Penfield and Boldrey, 1937). All subjects also had electrodes in the superior temporal region, known to engage in domain-general acoustic processing (Steinschneider et al., 2013). To disentangle the neural effects underlying linguistic processing from those related to linguistically non-specific phenomena, we gathered a set of parameters relevant to the description of words/clauses with regard to their duration and position in the stream of speech. We collected three duration-related parameters: the duration of the word in ms (ws_we), the duration between word start and speech start in ms (ss_ws), and the duration between word end and speech end in ms (we_se). These were obtained by calculating the temporal distance from ws to the closest ss, and from we to the closest se after completion of the aforementioned procedures for tag validation and semi-automated correction. The same was done for the starts and ends of the simple clauses, whereby cs_ce, ss_cs, and ce_se were calculated.

To evaluate the potential interference of EMG activity with our findings, we accounted for the available EMG recordings. The EMG recordings analyzed consisted of one EMG electrode on the left cheek in S3 and S4, two electrodes from the bilateral quadriceps plus two electrodes from the bilateral deltoid muscles in S5, and two bilateral EMG electrodes in the upper chest region of S1. The electrodes in the latter subject lay in the approximate area of the muscle trapezius, which is involved in arm movements. The EMG levels S1 thus likely reflect myographic activity of the upper extremities ipsilateral to the positions of these electrodes. No EMG data were recorded in S2. We analyzed the EMG data in the frequency range of 60–200 Hz, since EMG activity in these frequencies was the most pronounced. The RSM of the EMG responses was calculated in the same way as for the neural data and averaged over 60–200 Hz and over the duration of the respective word. The RSM values obtained were trial-averaged and rendered on a natural logarithmic scale.

Another linguistically unspecific parameter we wanted to control for was the intensity (volume) of the acoustic signals underlying word production. We obtained this information from the wav data recorded concurrent with the ECoG signals while the patients were speaking. This was done by automated identification of the transcribed words in the acoustic signals and by subsequent manual correction of the resulting annotations. The transcribed linguistic material was pre-processed using custom-made Python-based software (courtesy of Benedikt Sauerborn) to ensure the compatibility of our data with the software applied subsequently to automatically align the transcribed words to the acoustic signal. Word-for-word segmentation of the acoustic signal was performed using the Munich Automatic Segmentation System MAUS (Kipp et al., 1997; Schiel, 2015). The temporal precision of this alignment was checked and improved when necessary by manual inspection of the segmented data using PRAAT. Average (mean) values over the duration of each word for the intensity of the acoustic signals in Db were obtained.

These and some other linguistic parameters were used to identify the collinearity structure in our linguistic data and accounted for in word-level correlation analyses of linguistic parameters with the neural activity.

### Calculation of Number of Spoken Syllables in a Word and Consonant-to-Vowel Ratio

Using the phonological information, we generated two additional parameters which are relevant to description of articulatory complexity. We calculated NoS and CVR (i.e., the ratio of consonants to vowels in a word). The latter was calculated by dividing the number of consonants by the number of vowels in a spoken word.

### The Articulatory Complexity Index

We estimated the articulatory complexity of the words using a mathematical model by Ziegler and Aichert (2015). Based on the annotations of the consonant cluster structure, syllabic composition, and the prosodic properties of the words, we calculated EoA with the help of a custom-made MATLAB-based program (Supplementary Appendix 1.3). A high EoA index reflects greater ease of articulation and a low EoA index is a sign of high articulatory complexity.

### Correlations Between the Linguistic Parameters

Each of the linguistic parameters (i.e., FRQ, EoA, CVR, NoS, ss_ws, ws_we, we_se, EMG levels, and the intensity of the acoustic signal) in every subject was concatenated for all PoS of the words selected as for the underlying neural data. Custom-made MATLAB-based software was used to make sure that the temporal arrangement of the linguistic and the corresponding neural data was exactly the same. Next, these parameters were correlated with each other. All linguistic parameters were rendered on a natural logarithmic scale, before which 0.001 was added to each value to avoid rendering all zeros to minus infinity (see Solari, 1969 for methodological considerations). We used Pearson’s correlation implemented in the built-in MATLAB function corr.m and tested the *r* values obtained for significance at *p* < 0.05. The results of this analysis were used to assess the reproducibility of the correlation structure between the parameters, and they were taken into account when interpreting the results of correlation-based neural analyses.

### Correlations of Relative Spectral Magnitudes With the Linguistic Parameters

**FIGURE 3:**
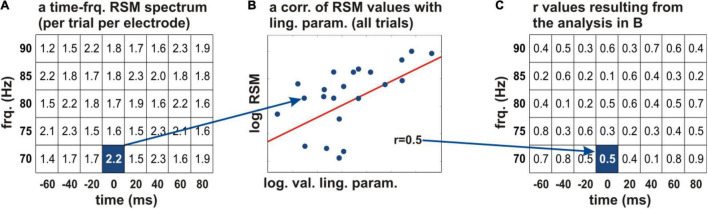
Correlation of the neural activity with the acquired linguistic parameters. **(A)** The logarithmic relative spectral magnitude (RSM) values at an individual electrode are depicted schematically in every time-frequency bin of the spectrum (the squares of the table) for a frequency range of 70–90 Hz (y axis) and −60 to 80 ms relative to word start (x axis). A Pearson’s correlation of the RSM values with a linguistic parameter of interest was calculated across all words of the given subject **(B)**. The correlation coefficients were projected on the time-frequency structure of the original spectrum **(C)**. This procedure allowed us to identify the time periods and frequencies in which the linguistic parameters showed correlations with the neural activity. The *r* values were color-coded and tested for significance. frq., frequency; log., natural logarithm; RSM, relative spectral magnitude; val., values; ling. param., linguistic parameters.

To identify the neural effects related to linguistic complexity, we correlated the individual parameters with the neural activity. The parameters we were investigating present data distributions which do not fall into discrete natural categories, so we chose this procedure over group comparisons (Baayen, 2008). We will further refer to this procedure as “neurocorrelation.” Figure 3 explains the principle of this analysis. Its results were visualized by projecting the obtained *r* values on the original time-frequency space of each electrode and color-coded for the strength and prefix of the correlation. Prior to correlation, the RSM values and all linguistic parameters were rendered on a natural logarithmic scale.

We first conducted the neurocorrelation analysis for the frequencies up to 300 Hz over the entire time window of 2 s before and 3 s after word onset. Correlations of RSM values with the linguistic parameters showed effects which were more local in time and frequency than those of the duration-related parameters. They showed no clear patterns in very high gamma frequencies over 150 Hz nor at very early and late time points of the time window analyzed. To decrease the number of multiple comparisons for statistical analyses and to focalize the neurocorrelations in the time and frequency space, we repeated the same analysis for all parameters in frequencies up to 150 Hz, which is a commonly used upper limit for time-frequency analyses of speech-related gamma activity in ECoG studies (e.g., Sinai et al., 2005; Towle et al., 2008; Derix et al., 2014). We shortened the time window analyzed to the period from 500 ms before ss to 500 ms after se for the same reason.

Statistical testing was conducted using Bonferroni correction for multiple comparisons over all time-frequency bins and electrodes at *q* < 0.05. Bonferroni-corrected effects will be reported wherever they could be observed. The results of uncorrected testing will be reported otherwise. When applying statistical testing on the correlations of the RSM values with the individual linguistic parameters, we noticed that while some parameters, such as the duration-relevant ones, yielded very strong effects which survived conservative statistical testing, others yielded significant effects only using less conservative thresholds, if any effects for the given parameter could be observed at all. This is not surprising, since the parameters investigated describe different aspects of the linguistic data which may be differently represented in neural activity. If one selects a single conservative test with a conservative threshold, one may overlook effects related to some parameters of interest. On the other hand, however, if one tests all data using a non-conservative statistical approach, this may compromise the spatial specificity of the neural effects and produce a topographically meaningless picture. Due to these considerations, we applied a sequence of tests which were either more or less conservative and reported the most conservative test and threshold per parameter and subject that was showing significant effects. Note that, for the sake of the statistical robustness of the results, we considered the uncorrected effects as “significant” if the *p*-values did not exceed 5E-06. All effects with higher *p*-values were treated as not significant. We tested the neurocorrelation effects using a scale of significance thresholds ranging from 5E-06 (the least conservative) in steps of 5E-06 until no effects related to the given parameter could be observed (Supplementary Appendix 2.2). The last threshold yielding significant effects for the given parameter and subject will be reported.

We accounted for the occurrence of potential mutually confounding effects, i.e., the effects of several parameters observed when testing the neurocorrelations for significance at the same electrode, in the same time range (a maximum temporal distance between effects was 40 ms) and in the same frequency range (a maximum difference in the frequency of the effect was 20 Hz within gamma frequencies over 35 Hz or within the same frequency band in the alpha (5–10 Hz) and beta (15–30 Hz) signal components, see Supplementary Appendix for details on the latter two frequency ranges). We defined “the same” temporo-frequential components as intervals rather than as single time-frequency bins so as not to overlook the overlaps between parameters which would have been evident when testing the parameters using less conservative significance thresholds.

**FIGURE 4:**
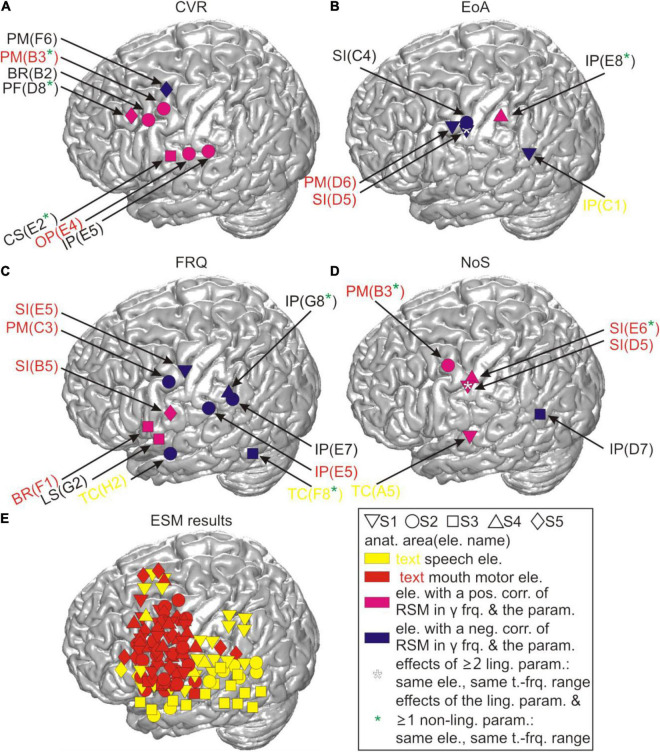
Correlations between RSM responses and the linguistic parameters before residualization in relation to cortical anatomy. All locations of the effects in gamma frequencies are visualized on the cortical surface based on their MNI coordinates for **(A)** CVR, **(B)** EoA, **(C)** FRQ, and **(D)** NoS. CS, central sulcus; LS, lateral sulcus. **(E)** The anatomical locations of all potentially speech-relevant electrodes are visualized using the same procedure as in **(A–D)**. anat. area(ele. name), anatomical area (electrode name); pos./neg. corr., positive/negative correlation; y frq., gamma frequencies (40–150 Hz); param., parameter; ling., linguistic; t.-frq. range, time-frequency range; BR, Broca’s area; other abbreviations as in Figure 1A.

**FIGURE 5:**
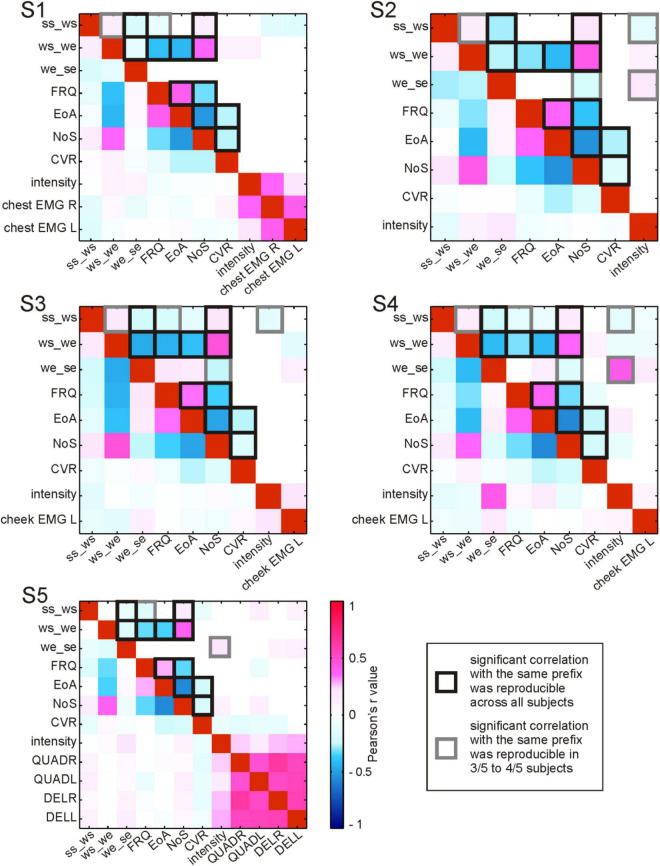
Correlations between parameters accompanying the production of content words. For each subject (S1–S5), Pearson’s r correlation values between pairs of parameters are color-coded (see legend). Correlation values without statistical testing for significance are shown below the diagonal of red squares (a perfect positive correlation of each parameter with itself); above the diagonal: significant (<0.05) correlations. ss_ws, the duration from speech start to word start in ms; ws_we, the duration from word start to word end in ms; we_se, the duration from word end to speech end in ms; FRQ, lemma frequency extracted from the linguistic corpus (FOLK, 2012); EoA, ease-of-articulation index (Ziegler and Aichert, 2015); NoS, number of syllables in a spoken word; CVR, consonant-to-vowel-ratio (the number of consonants in a spoken word divided by the number of vowels); intensity, average intensity of the acoustic signal during word production in Db; EMG, average relative spectral magnitude values for electromyographic activity during word production recorded at the respective body part; L, left; R, right; QUADR/QUADL, EMG from the right/left quadriceps; DELR/DELL, EMG from the right/left deltoid muscles. The squares with black and gray outlines highlight the correlations that were reproducible across subjects.

Effects were indeed observed at the same electrode and in the same temporo-frequential components of the neural signal (Figure 4), and the linguistic parameters analyzed were often significantly correlated. Some neural effects describing a particular parameter may therefore have come into being due to collinearity. We were, however, interested in finding out to what extent parameter-specific effects could be identified. Therefore, we conducted an additional analysis to erase the mutually orthogonal components in the linguistic data. Using a linear regression lmer function implemented in the lme4 package (Bates et al., 2014) for R, we predicted each linguistic parameter by all parameters with which it was significantly correlated^1^ (Figure 5). By doing so, we were able to extract the residuals of the models, which were orthogonal to these parameters. In a next step, we correlated the residuals with the neural activity.

## Results

### The Selected Linguistic Material

Words no longer than three spoken syllables were used due to the constraints related to the calculation of EoA (Supplementary Appendix 1.3). We were able to obtain sufficiently large data sets for statistical analysis in spite of this constraint (cf. Kern et al., 2019). The numbers of tokens per subject was 65–156 for the category ADV, 115–263 for FV, and 60–177 for NN (see Supplementary Appendix 2.1 for additional details).

### Typical Relative Spectral Magnitudes Changes Related to Word Production

We conducted a spectral magnitude analysis to find out if the gathered content words would be associated with focal and reproducible patterns of neural activity. The RSM spectra, triggered to word onset and averaged over the entire number of content words in each subject, were calculated and tested for significance (Figure 1B).

The most pronounced neural responses occurred in a broad range of gamma frequencies, in which they were manifested as increases in the spectral magnitude (Figure 1B). These responses were observed at individual electrodes on the pericentral mouth motor cortex, and they were significant not only during but also before and after the onset of word production. These effects tended to start around speech start and end between word end and speech end. This suggests that they contributed to preparatory and executional processes. More attenuated changes in gamma activity occurred at other cortical locations including the posterior part of Broca’s area adjacent to the motor cortex and in the superior temporal and parietal regions. Since all words in this analysis were trial-averaged regardless of their linguistic properties, these responses likely reflect general, linguistically unspecific articulatory features related to word and speech production.

### Correlation Structure in the Linguistic Data

The evaluation of collinearity between the linguistic parameters revealed a number of statistically significant correlations which were reproducible across subjects (Figure 5). These correlations will be referred to as “strong” whenever the *r* values exceeded 0.4 and as “weak” otherwise.

Among the linguistic parameters, EoA, for which higher values indicate greater ease of word production, consistently showed strong positive correlations with FRQ. This means that, with the help of Ziegler and Aichert’s model (2015), frequent words were modeled as easier to articulate than rare words. The inverse relation between word frequency and complexity in our data is in line with psycholinguistic findings that high-frequency words are recognized and named faster and that they are more often pronounced correctly in comparison with low-frequency words (Oldfield and Wingfield, 1965; Connine et al., 1990; Bose et al., 2007). The strong negative correlation of EoA as well as FRQ with NoS also indicates that more frequent words, which were associated with greater ease of articulation, also tended to have fewer syllables. The strong negative correlations between both EoA and FRQ with word duration (ws_we) showed that the production of articulatorily complex, low-frequency words lasted longer. As one would expect, NoS was strongly positively correlated with ws_we. The negative correlation between lexical frequency and these measurements of word length is in line with the observation by Zipf (1935, p. 38) that “the length of a word tends to bear an inverse relationship to its relative frequency.” CVR was weakly negatively correlated with EoA, suggesting that consonants made words more difficult to pronounce than vowels did. This agrees with the linguistic and with the psycholinguistic literature indicating that the articulation of consonants is more demanding than the articulation of vowels (Ziegler and Aichert, 2015). A weak negative correlation between CVR and NoS occurred in all subjects. The words with more syllables thus tended to have proportionally fewer consonants than vowels compared with shorter words. This is not surprising, considering that vowels are essential building blocks of syllables.

We made an interesting observation with respect to a word’s position in the speech production epoch. In S1–S4, the duration from articulation onset to word start (ss_ws) was weakly positively correlated with ws_we. Similarly, the duration from word end to speech end (we_se) in all subjects showed weak to strong negative correlations with ws_we. Longer words were thus farther away from ss and closer to se than shorter words. ss_ws showed reproducible weak negative correlations with FRQ (S1, S3–S5) and a weak positive correlation with NoS (S1–S5). This not only mirrors the aforementioned inverse relation between NoS and FRQ but it also indicates that low-frequency, polysyllabic words tend to have a greater temporal distance to ss. At the same time, the weak negative correlation between we_se and NoS (S2–S4) suggests that the duration we_se is shorter for words with more syllables. Taken together, these correlations provide evidence that longer and less frequent words have a tendency to occur later in the speech production epoch. This agrees with previous findings that high-frequency, shorter words display a tendency to occur earlier in multi-word sequences during speech production, likely because they are easier to access in the mental lexicon than low-frequency, longer words (Keenan and Comrie, 1977; Kelly et al., 1986; Navarrete et al., 2006).

Interesting reproducible correlations were observed for two duration-related parameters with the intensity of the acoustic signal. It was weakly negatively correlated with ss_ws (S2–S4) and weakly to strongly positively correlated with we_se (S2, S4–S5). Accordingly, words which occurred earlier in a speech production epoch had a tendency to be pronounced more loudly. Reproducibility with regard to correlations of the linguistic parameters with EMG activity could not be assessed, since the locations of the EMG channels differed between the subjects.

The reproducible correlations between EoA, NoS, CVR, FRQ and the duration-related parameters were consistent with psycholinguistic literature. They reflected the organizational properties pertinent to human language as a compositional, rule-based system. The presence of multicollinearity (Figure 5) described here was taken into account in the subsequent analyses aimed to identify parameter-specific effects in the neural signals.

### Neurocorrelations of Relative Spectral Magnitudes Changes With Duration-Related Parameters

**FIGURE 6:**
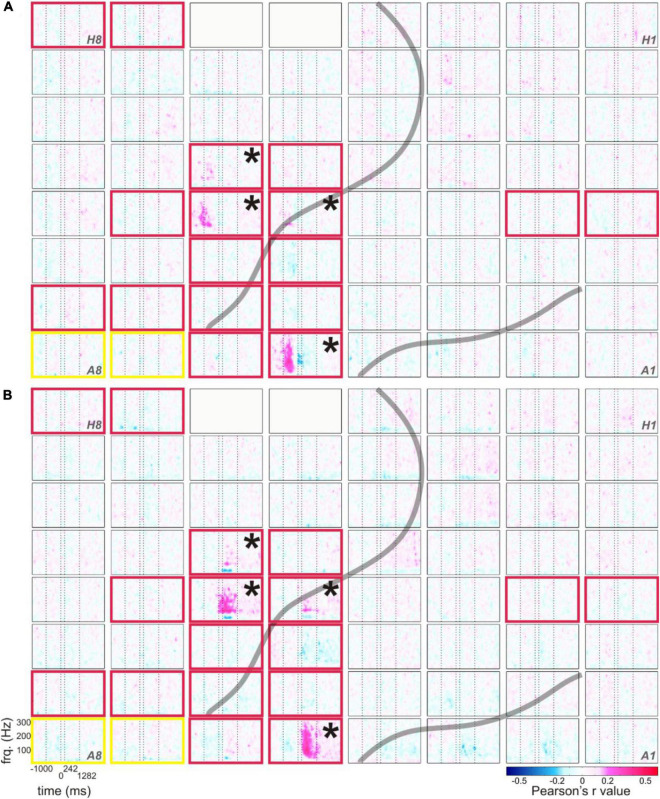
Typical correlations of RSM responses related to the production of content words with the duration from speech start to word start **(A)** and with the duration from word end to speech end **(B)**, example from S5. The correlation values of RSM with the respective parameters are color-coded (see legend), all other conventions as in Figure 1B.

Out of all linguistic parameters which we correlated with the RSM values, those related to the position of the word within a speech production epoch elicited most pronounced neurocorrelation patterns. These are illustrated in Figure 6 (S5). This figure shows neurocorrelation results for the parameters ss_ws and we_se in the panels A and B against the individual structural and functional anatomy of the subject. The effects representative of these parameters were spatially focalized to electrodes within the pericentral mouth motor cortex. They occurred at the same electrodes for both ss_ws and we_se, and also at the same electrodes at which the clearest RSM responses underlying the production of content words could be observed (Figure 1B). The presence of such correspondence supports the suitability of the neurocorrelation approach for identifying the neural signal components modulated by a given parameter.

As in the RSM analysis, most pronounced effects occurred in the gamma frequencies. Interestingly, the maximum spectral magnitude changes underlying the production of content words and the highest positive correlation values with the duration-related parameters ss_ws and we_se occurred at the electrodes at which the frequency range of the effect was broadest (cf. Figures 1B, 6). The gamma effects in the neurocorrelation analysis differed in the timing of their occurrence between ss_ws and we_se. They tended to occur prior to the onset of word production for ss_ws and mostly after word production for we_se.

In comparison with the parameters ss_ws and we_se, the neurocorrelations with word duration (ws_we) did not show such pronounced effects (data not shown). Although the tags for word and speech starts and ends were set according to the same principles, the neurocorrelation effects related to word duration were more attenuated, and they seldom reached significance in the Bonferroni-corrected testing for multiple comparisons over all time-frequency points and electrodes (*q* < 0.05). Generally, ss_ws showed most pronounced positive correlations with RSM values around speech start (before and after speech start but prior to the start of the word) and the strongest positive correlations with we_se occurred between word end and speech end.

### Statistical Testing of Neurocorrelation Results Prior to Residualization

Figure 4 visualizes the pre-residualization results of testing correlations between RSM responses and the linguistic parameters with the most conservative statistical test and threshold. The effects observed in relation to the four main linguistic parameters of our interest (i.e., CVR, EoA, FRQ, NoS) were observed in areas roughly corresponding to speech and mouth-relevant regions (Figure 4E). In agreement with our expectation that higher levels of gamma activity would be associated with increased word complexity, we observed that CVR mostly elicited positive correlations with high-gamma activity, EoA and FRQ yielded effects which were predominantly negative, and NoS mostly showed positive effects. These effects did not occur over broad ranges of gamma frequencies or over extended time periods (Supplementary Appendix 2.2), in contrast to the correlation effects observed with the linguistically unspecific parameters related to speech duration (Figure 6).

### Statistical Testing of Neurocorrelation Results After Residualization

As mentioned above, the linguistic parameters were often correlated with each other, e.g., EoA was negatively correlated with NoS in all subjects (*p* < 0.05, uncorrected). As a result, the outcome of the neurocorrelation analysis with an individual linguistic parameter may produce effects which are not specific to the given parameter but partially reflect the fact that this parameter is correlated with another parameter and that this other parameter plays a role. In an attempt to draw parameter-specific conclusions, we conducted a linear regression analysis and extracted its residuals, representing mutually orthogonal components. Then, we repeated the same neurocorrelation analysis with the residuals and compared the resulting neural effects with those that we observed prior to residualization. Our post-residualization analysis yielded significant effects for two of the parameters investigated.

**FIGURE 7:**
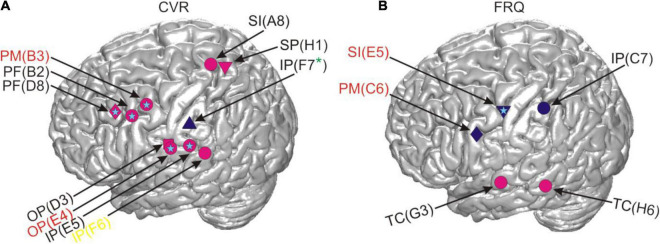
Correlations between RSM responses and the linguistic parameters CVR and FRQ after residualization in relation to cortical anatomy. The light blue stars indicate that the same effect was also present in the neural data prior to residualization and the absence of such a star indicates the contrary. All other conventions as in Figure 4.

Figure 7 shows the locations of the results for CVR and FRQ we observed in post-residualization neurocorrelation analysis (Supplementary Appendix 2.2). The neurocorrelation effects related to CVR survived residualization and occurred in the same time and frequency ranges (see section “Materials and Methods”) and at the same electrodes and with the same prefixes in two subjects. Two major anatomical sites at which these effects were observed can be distinguished: the border of the prefrontal and premotor cortex and the parietal operculum converging on adjacent other parts of the inferior parietal cortex (Figure 7A). This anatomical picture is very similar to the one observed prior to residualization (Figure 4). Several other effects are new and it is hard for us to interpret their location and functional significance due to the relatively small sample of subjects. The effects of FRQ in the fronto-parietal cortex were less conclusive: only one electrode in the primary somatosensory cortex of one subject showed an effect at the same time and frequency range and with the same correlation prefix. The correlation effects related to this parameter prior to residualization mostly occurred in the fronto-parietal cortex with a negative correlation prefix (Figure 4C). This was also the case with post-residualized data in these anatomical areas (Figure 7B). In addition to these effects, positive correlations with high-gamma frequencies which had not been visible in the pre-residualized data could be observed in one subject.

## Discussion

The linguistic complexity of words has largely been studied on the behavioral level and in experimental settings. Only little is known about the neural processes underlying this phenomenon in uninstructed, spontaneous conversations. We used ECoG recordings from the fronto-temporo-parietal cortex of five epilepsy patients to investigate how the linguistic complexity of content words is reflected in cortical activity obtained during real-life speech production. We took an integrative approach involving different measures of word complexity. The parameters investigated were (i) the number of spoken syllables in a word (NoS), (ii) the consonant-to-vowel ratio (CVR) calculated by dividing the number of consonants by the number of vowels in the spoken word, (iii) the “ease-of-articulation” (EoA) index calculated according Ziegler and Aichert’s model (2015), and (iv) the lemma frequency of content words (FRQ).

We performed time- and frequency-resolved correlations of the linguistic parameters with word-accompanying RSM data (Figure 4). Considering that many parameters turned out to be correlated with each other and with potentially confounding control variables such as word or speech duration (Figure 5), we orthogonalized the parameters by extracting residuals with the help of a linear regression model. After this, we repeated the correlation analysis with the residuals for each of the linguistic parameters (Figure 7). The effects we were able to observe when correlating the RSM data with word-complexity measures occurred, as expected, predominantly in gamma frequencies. Increased activity in gamma frequencies can be associated with higher effort (Senkowski and Herrmann, 2002). High values for the parameters NoS and CVR and low values for the parameters EoA and FRQ are associated with high word production effort. We were therefore expecting positive correlations of gamma activity with the former two parameters and negative correlations with the latter two parameters. The correlation effects were, indeed, in agreement with our expectations: prior to residualization, CVR and NoS yielded positive (CVR) or mostly positive (NoS) correlations with gamma activity, and EoA and FRQ yielded predominantly negative correlations (Figure 4). This prefix tendency was mostly maintained, whenever significant neurocorrelation effects could be observed after residualization (Figure 7). We were able to identify CVR (the proportional relation between vowels and consonants) as the most informative parameter showing meaningful correlations with high gamma activity at the junction of the premotor cortex with BA 44 and the prefrontal cortex, as well as in the ventral postcentral region. These effects occurred before and after residualization, which may be an indication of their higher robustness compared to the other parameters (cf. light blue stars in Figures 4, 7). In terms of frequency and time, however, they were less reproducible (Supplementary Appendices 3.2, 3.3). This may indicate that the linguistic parameters studied are only moderately represented in the neural signals investigated. Since the spectrum of gamma frequencies is a composite phenomenon which relies on multiple cell types (Buzsáki et al., 2012), however, it is also conceivable that these temporo-frequentially narrow effects may indicate the functionally specific activation of narrow populations of neurons whose signal properties vary between linguistic parameters and subjects.

Previous lesion-based and functional magnetic resonance imaging studies based on lexical decision tasks attribute dissociations between consonants and vowels to the differential demands on the processing of the lexico-semantic and prosodic aspects of speech (Caramazza et al., 2000; Carreiras and Price, 2008). Caramazza et al. (2000), for instance, suggest that the independent neural representation of consonants and vowels could serve as the basis for the assignment of segments to nucleus and non-nucleus positions in a syllable, and Carreiras and Price (2008) attribute neural differences between consonants and vowels to different attentional demands during processing. The facts that the most robust effects related to CVR were observed in higher-order areas such as the parietal and the dorso-ventral prefrontal cortex (Figure 7) and that these areas did not show mouth motor effects during ESM (Supplementary Table 7) are in line with the idea that the CVR-related effects may reflect higher-order processes involved in the monitoring and segmentation of speech. Based on our data, we find it difficult to speculate on why this parameter yielded more reproducible effects after residualization than others. This may show its greater salience in the neural signals or suggest that the other parameters have stronger representations outside of the anatomical areas our surface grid electrodes can capture signals from. The anatomical location of the CVR-related effects agrees with the location of areas which were most informative about the distinction between consonants and vowels in the single-trial-decoding study by Pei et al. (2011). This similarity indicates that spontaneous and experimentally elicited speech involve anatomically similar neural resources, at least regarding the production of vowels and consonants. It also shows that the phonological composition of speech is a promising way to understand the functional organization of the language-relevant pericentral cortex (Blakely et al., 2008; Bouchard et al., 2013; Mugler et al., 2014; Ramsey et al., 2018).

FRQ and NoS showed effects in the temporal cortex or on the lateral sulcus, and one subject had an effect in Broca’s area in relation to FRQ. These areas are known to be language-relevant (Hickok, 2012), and their contribution to word complexity-related processes is plausible. With the exception of the parameters EoA and NoS, which had a shared effect at the same electrode lying in the primary somatosensory cortex and also within the same time-frequency range (marked by a white star in Figure 4), the effects observed occurred at different electrodes. That EoA and NoS had a shared effect was not surprising, given that syllable-structure information contributed to the calculation of EoA (Supplementary Appendices 2.2, 2.3). After residualization, most of the effects related to these three parameters disappeared, which may be an indication of their weak robustness, or also a reflection of the fact that the removal of collinearity may have left too little meaningful information in the residuals, especially from the parameter EoA, which is composed of multiple articulation-relevant aspects.

Lexical frequency did not show the clearest effects in comparison with other parameters. Besides the strong effects of the temporal distance between speech and word start as well as speech and word end along the course of the central sulcus (Figure 6), it was the CVR that yielded most robust results that could be observed in correlations of this parameter with the neural signal, both before and after residualization (Figures 4, 7). The fact that the negative correlations of FRQ with cortical activity that were observable with high gamma activity, which was in agreement with our hypothesis, could be observed prior to but not after residualization, may be related to the presence of strong correlations between this parameter with other parameters of the linguistic data (Figure 6 and Baayen, 2010). The extent to which variance in lexical frequency can be explained by other linguistic factors is an interesting question for investigation which may further extend current understanding of this phenomenon. With regard to our scarce findings on the neural representation of word frequency, a comparison with a previous study from our lab is possible. In her doctoral study based on our corpus, Diekmann (2019) arrived at the same conclusion, although she used a different approach to speech segmentation (automated and manually corrected setting of word borders in the acoustic data with *post hoc* co-registration with the ECoG signal) and implemented a different corpus for the extraction of lexical frequencies (Faaß and Eckart, 2013). We thus think that it is unlikely that the limited evidence pointing to clear effects of lexical frequency in our spontaneously spoken data can be attributed to errors in speech segmentation and frequency assignment.^2^ It is conceivable that data from a larger cohort of subjects and from additional brain areas may yield a clearer picture than in our data. Addressing this question with the help of ECoG, however, will require larger datasets than those currently available in the FNLC.

From a linguistic point of view, our study is in line with previous work describing linguistic complexity as a phenomenon with multiple instantiations (e.g., Miestamo, 2006a,b). In spite of the presence of collinearity between the linguistic parameters used to assess word complexity in our data (Figure 5), the parameters were not mutually redundant. The neural effects we have observed indicate that not any parameter describing word complexity shows effects in the same anatomical area, at the same statistical threshold or at identical time points. These physiological observations support the idea that “word complexity” cannot be reduced to a single aspect of speech but that taking its multiple aspects into account is necessary to obtain a comprehensive understanding of this holistic phenomenon and of the neural infrastructure behind it (Ziegler and Aichert, 2015).

Our data were collected under conditions of spontaneous, uninstructed communication. This choice of data was motivated by concerns of ecological validity of the spoken material and of the associated neural recordings. Several challenges one has to meet when dealing with such data, however, need to be taken into account. First, ECoG data emerge as a by-product of pre-neurosurgical evaluation, which is carried out in comparatively few institutions in Europe, and such recordings are relatively rare. This inevitably limits the sample of subjects that can be studied.^3^ Second, the presence of collinearity between linguistic parameters, as well as possible contributions of EMG and auditory signal properties to the neural effects observed need to be taken into account (Figure 5). While we were able to account for a number of variables, the influence of other factors, such as medication or some additional aspects of behavior that have not been captured, cannot be excluded. The experimental approach has an important advantage inasmuch as it enables rigid control of potentially confounding factors, which may not always be possible in non-experimental studies. By placing the focus of the present research on natural communication, we do not seek to question the importance of previous evidence obtained using experimental psycho- and neurolinguistic research. Instead, we believe that systematic comparisons of results obtained using both approaches can lead to a better understanding of the neural and behavioral processes that are typical of human communication.

## Conclusion and Outlook

In this study, we undertook an attempt to elucidate the neural correlates of processing related to word complexity under conditions of experimentally unconstrained, real-life speech production. We correlated several measures of word complexity—namely, length in syllables, CVR lexical frequency, and a composite measure of articulatory movement factors designed to express “ease of articulation” (Ziegler and Aichert, 2015)—with ECoG data underlying the naturalistic, uninstructed speech of five epilepsy patients. Out of the parameters investigated, we were able to identify CVR as the parameter showing most reproducible effects. These could be localized to two areas: the frontal one was at the junction of the premotor cortex, the prefrontal cortex, and Brodmann area 44, and the postcentral one lay directly above the lateral sulcus and comprised the ventral central sulcus, the parietal operculum and the adjacent inferior parietal cortex.

In addition to the inevitably small number of ECoG-implanted subjects available for such research, an important methodological challenge dealt with the question of how to extract controlled material out of the variable and complex behavioral and linguistic data representative of natural verbal communication. We show how it is possible to achieve control over numerous potentially confounding variables and to obtain meaningful neural effects from ECoG recordings underlying spontaneously spoken language. These observations speak for the plausibility of the non-experimental approach (Derix et al., 2012, 2014; Ruescher et al., 2013; Wang et al., 2016) as a potentially worthwhile complement to experimental work, and they open up interesting and still little-explored possibilities to study the neural correlates of linguistic processing during real-world verbal interaction.

## Data Availability Statement

The data and the code that support the findings of this study are available from the corresponding authors upon reasonable request.

## Ethics Statement

The studies involving human participants were reviewed and approved by the Ethics Committee of the University Medical Center Freiburg. The patients/participants provided their written informed consent to participate in this study.

## Author Contributions

All authors listed have made a substantial, direct, and intellectual contribution to the work, and approved it for publication.

## Conflict of Interest

The authors declare that the research was conducted in the absence of any commercial or financial relationships that could be construed as a potential conflict of interest.

## Publisher’s Note

All claims expressed in this article are solely those of the authors and do not necessarily represent those of their affiliated organizations, or those of the publisher, the editors and the reviewers. Any product that may be evaluated in this article, or claim that may be made by its manufacturer, is not guaranteed or endorsed by the publisher.

## Footnotes

1 We predicted each parameter of interest only by the parameters with which it was significantly correlated and not by all other parameters in order to not erase too much variance in the linguistic data in the process of residualization. Using all parameters as predictors could have resulted in losing potentially meaningful information.

2 Also note that we have implemented extensive checking procedures to prevent errors in annotation and co-registration of materials from different modalities (see section “Materials and Methods”).

3 In comparison with non-invasive methods, ECoG studies usually investigate smaller populations. Note that the sample size in our work is not uncommon in ECoG research: Crone et al. (2001a,b), e.g., investigated high-gamma activity patterns during speech production in one (2001a) and during speech perception in four subjects (2001b), Bouchard et al. (2013) studied the functional organization of the sensorimotor cortex during speech articulation in three subjects, and Herff et al. (2016), who attempted to reconstruct speech from ECoG activity recorded from temporal areas during speech perception, report on results from a single subject. Our cohort of five subjects thus falls within the scope of a usual size for ECoG-based language studies. Comparisons of our findings with results obtained using other, non-invasive methods, as well as validation of our findings by follow-up ECoG studies into the neural representation of natural language will certainly be of interest.
