# Supplementary material for: A Study of Word Complexity Under Conditions of Non-experimental, Natural Overt Speech Production Using ECoG
Source: Front Hum Neurosci. 2022 Feb 4;15:711886. doi: 10.3389/fnhum.2021.711886 (PMC8854223; doi:10.3389/fnhum.2021.711886)
Supplement: Supplementary file 1 [file Data_Sheet_1.PDF]

Appendix

1. Supplementary methods

1.1. Transcription of the spoken material

Trained linguists performed transcriptions of the patients' speech using the freeware PRAAT (Boersma 2001) in accordance with the "basic" transcript of the GAT-2 conventions (Selting et al. 2009). This transcription method allows segmenting continuous speech in individual IPs, which are characterised by its cohesive intonation contour and meaning. These conventions allow annotation of both what and how is being said. The latter includes the placement and prosodic prominence of the focus accent (the whole syllable is annotated in capital letters) and of secondary accents, pause durations, suprasegmental (e.g., the IP-final falling intonation is annotated by a semicolon), and some paralinguistic properties. Examples of IPs are provided in Example 1. This method of transcription allows documenting colloquial, idiolectal, and dialectal properties of language such as elisions and other deviations from the standard German. It is therefore well suited to provide realistic descriptions of natural speech without losing information about such properties. The high precision of annotation, however, comes at a cost of time. In our experience, the average transcription rate was approximately one min. of continuous speech per 45 min. for an expert and around one hour for transcribers with less experience. All transcriptions were checked by at least two other linguists, who cross-validated the borders of the IPs and the linguistic annotations within them. If differences between the transcriber and the cross-validators and/or among the cross-validators with regard to the content and/or the position of the primary accent of the IP were identified, the IP was excluded from neurolinguistic analyses in favour of unambiguous language material. Little spoken material had to be discarded at this step of data acquisition owing to the pre-selection of high-quality language material at a previous stage of data acquisition.

German transcription	German standard spelling	English translation
das HEIßT-	Das heißt,	That is,
=sie haben jetzt praktisch (.) gestern so ne art LANDkarte (.) beim gehirn aufgeschrieben;	sie haben jetzt praktisch gestern so eine Art Landkarte beim Gehirn aufgeschrieben.	they actually wrote down some kind of a brain map yesterday.
aber jetzt können sie dann noch nicht SAgen-	Aber jetzt können sie dann noch nicht sagen:	But they still cannot tell yet,
=ach da verMUten wir was-	Ach, da vermuten wir was.	Oh, we suspect something here.
=da ISCH jetz:-	Da ist jetzt	This is now
DER nerv oder DIE funktion;	der Nerv oder die Funktion.	this nerve or that function.

Example 1: A typical transcription excerpt. Syllables in capital letters highlight the focus accent. "-" indicates no change in IP-final intonation, "=" indicates a smooth, immediate acoustic transition between adjacent IPs, "(.)" annotates pauses <200 ms, square brackets indicate speech excerpts in which the speaker overlapped with at least one of his/her conversation partners, and ";" is an index of IP-final falling intonation.

1.2. Definition and extraction of "simple clauses"

1.2.1. Extraction of simple clauses

A simple clause was defined as the main verb (the full verb, the copula verb, or a transitive modal verb in the absence of a full verb (Eisenberg 2004) plus all its arguments. The borders of the simple clauses were set in accordance with the topological field model of German utterances (Musan 2009). It defines discrete syntactic fields which occur in the following order: "Vor-Vorfeld" (VVF) containing lexical elements which have a loose syntactic relation to the main verb and which do not form arguments of the simple clause, "Vorfeld" (VF) containing lexical elements which form sentence constituents such as subjects, objects, or adverbial modifiers and their integral parts, "Linke Klammer" corresponding to the finite verb of the main clause or to a subordinating conjunction of the subordinate clause, "Mittelfeld" containing lexical elements after the finite verb and until the end of the clause or until "Rechte Klammer" (RK) whenever it is present; RK corresponding to the non-finite verb; "Nachfeld" (NF) containing lexical elements which form sentence constituents such as post-positioned objects or adverbial modifiers which can otherwise also occur in VF; "Nach-Nachfeld" containing lexical elements which have a loose syntactic relation to the main verb, do not form arguments of the simple clause, and can otherwise also occur in VVF. All lexical elements between the start of VF and the end of NF were considered as falling within the borders of a simple clause (Musan 2009). Only clauses which were free of hesitation markers, self-corrections, stuttering, and consecutive repetitions of the same word were selected. All clauses which entered our selection contained a finite verb. Since psycholinguistic research has shown that humans perceive pauses ≥200 ms as actual "pauses" in a conversation (Walker and Trimboli 1982), we selected clauses with such pauses for the sake of homogeneity of our linguistic materials. The simple clauses obtained as described above form basic multi-word units of our neurolinguistic corpus which is currently being used by several projects at our lab which are dedicated to the neural architecture supporting linguistic functions.

1.2.2. Rendering the clauses in standard German

Next, the simple clauses from the IPs were rendered in standard German in accordance with the Duden online dictionary. We maintained the proximity to the spoken German as long as a word form was registered in Duden. Some German adverbs, for instance, 'heut'/ 'heute' (Engl. 'today') have parallel registered variants. Whenever our subjects said 'heut' or 'heute,' we differentiated between them. Phonological variants for endings of finite verbs in the first-person singular are also admissible in modern grammars, e.g., on Netzwerb (online). We therefore retained the presence or absence of "e" at the end of verbs in this form, e.g., 'ich sag'/ 'ich sage' (Engl. 'I am saying'). German has verbs written together with a detachable verbal particle corresponding to a separate adverbial modifier in English. There are no universal grammatical rules as to which non-finite verbs have to be written together with the counterparts of the English adverb and which not, and parallel forms are often registered in Duden. For instance, while 'danebenstehen' (Engl. 'to stand next to') is written together as

one word, ‘da sein’ (Engl. ‘to be here’) is written separately, and two parallel forms are registered in Duden (as of 2017) for ‘leer trinken’/ ‘leertrinken’ (Engl. ‘to drink empty’). We always kept to Duden whenever it offered one variant, and we opted for a composite form whenever two variants were listed as admissible. These phonological peculiarities have obvious consequences for linguistic analyses, and they had to be accounted for during the extraction of lemma frequencies.

Suppl. Fig. 1 provides as schematic overview of the linguistic procedures implemented from transcription to final word selection.

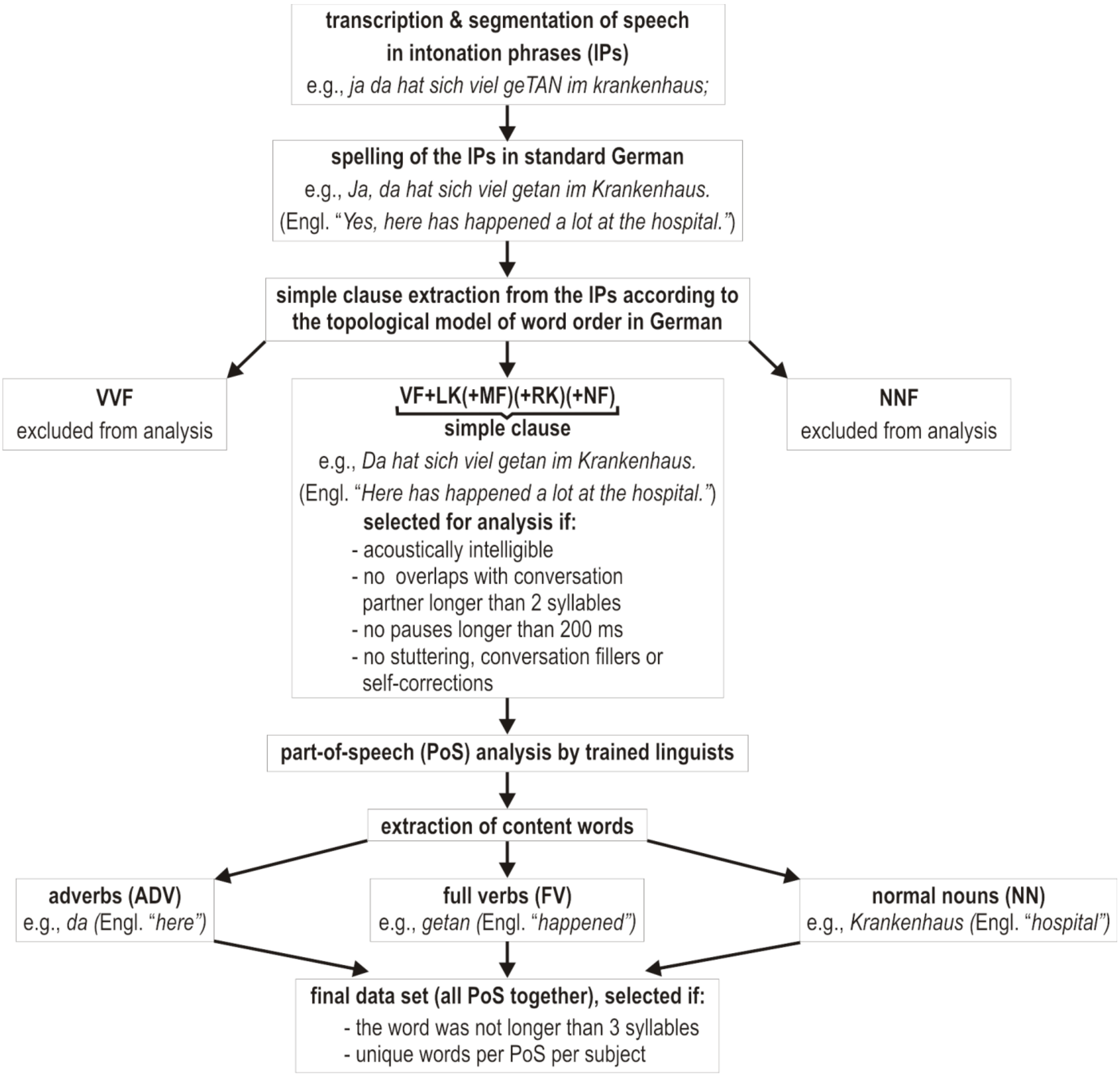

Supplementary Figure 1: A stepwise illustration of how the words for neurolinguistic analyses were acquired. Abbreviations: VVF: Vor-Vorfeld, VF: Vorfeld, LK: Linke Klammer, MF: Mittelfeld, RK: Rechte Klammer, NF: Nachfeld, NNF: Nach-Nachfeld. See Methods for details on these constituents of the topological model of word order in German.

1.2.3. Lemmatization and extraction of lemma frequencies

We used the Forschungs- und Lehrkorpus Gesprochenes Deutsch or FOLK (Engl. "Research and Teaching Corpus of Spoken German"), developed by the Institut für Deutsche Sprache (Engl. "Institute for the German Language" (FOLK 2012), to extract lemma frequencies.

This corpus consisted of 45,104 lemmata at the moment of data acquisition. Their lexical frequency was determined based on 1,308,786 word tokens (Dr. Thomas Schmidt, personal communication in 2015). An attractive characteristic of this corpus is that it provides PoS annotations of the word forms to each lemma in accordance with the STTS conventions. The lemma frequencies of the words in the patients' data were determined by searching for the lemma and the respective frequency in FOLK using a custom-made MATLAB-based program. The lemma frequency for the verbs was determined by accounting for the verb with a detachable verbal particle whenever the verb and its detachable verbal particle were produced together (e.g., in a non-finite verb form 'wahrgenommen' (Engl. 'perceived'). When the verb and the detachable verbal particle were produced separately (e.g., 'Sie *nimmt* das nicht *wahr*.' (Engl. 'She is not perceiving it.'), we used the frequency of the verb without a detachable verbal particle. The reason for this is that verbal particles in such cases obtained an own PoS tag according to the STTS conventions, just as all other words in our corpus did. Also, they are listed in FOLK with own lexical frequencies.

When the lemma from the patient data set was not found in FOLK, its frequency was first set to 0, and each lemma with this frequency was screened for correspondence between the lemmatization approaches of WEBLICHT and FOLK. Whenever necessary, corrections of the lemma form and of the associated lemma frequency were undertaken manually. The PoS category ADV accounted for two special cases: for some adverbs, two parallel lemma forms were listed in FOLK, e.g., 'gern' and 'gerne' (Engl. 'gladly'), each of them with its own frequency. We treated them as variants of one lemma which was assigned the sum of both frequencies. Another peculiarity with regard to some adverbs was that some of our lemmata had no exact matches in the lexical inventory of FOLK due to the fact that FOLK did not allow for the absence of the final "e" in adverbs such as 'heut,' which is a registered word in Duden. Whenever a lemma frequency for an adverb could not be found, our custom-made MATLAB-based software for extraction of lemma frequencies searched for a variant with an "e" at the end of the word and selected the respective frequency.

1.3. Application of the model of articulatory complexity by Ziegler and Aichert (2015)

We estimated the articulatory complexity of the words using a mathematical model by Ziegler and Aichert (2015). This is a tree-structure model that describes the hierarchical embedding of vocal-tract gestures in single words. It was developed to predict the accuracy of word articulation in patients with aphasia of speech (AoS). Ziegler and Aichert (2015) calculated the likelihood of correct word articulation by accounting for the number of accurate articulations

out of the total number of articulations of a set of words in 33 AoS patients. They identified a set of linguistic parameters relevant to the accuracy of word production on several levels of linguistic abstraction: consonant clusters, syllabic and prosodic structure levels. Based on them, these authors constructed a non-linear regression model, and trained it in predicting the likelihood of correct word articulation. In a cross-validation trial with a different group of 40 AoS patients, the model was able to predict this likelihood with a high accuracy ($R^2_{adj.}$ = 0.67). Its application to estimate articulatory complexity is therefore plausible. AoS is “an impairment of the capacity to program the movements of the articulators for the purpose of speaking” (Ziegler, 2008, p. 269). One can therefore assume that the probability of correct articulation estimated with help of Ziegler and Aichert’s model, further referred to as the *ease-of-articulation index* (EoA), reflects the requirements of motor planning of words in speech production.

The model by Ziegler and Aichert (2015) builds on the theory of articulatory phonology (e.g., Ohala et al., 1986; Goldstein and Fowler, 2003). This theory sees vocal-tract gestures, or discrete actions of articulatory organs (the lips, the tongue, the velum and the glottis), as basic units of articulation that are combined during the production of segmental components of speech. Columns 2-6 of Suppl. Tab. 1 provide an overview of what articulatory organs are involved in the production of German consonants (summarized based on Kortmann (2005) and Ziegler and Aichert, (2015)).

Supplementary Table 1. Articulatory features relevant to the production of German consonants. Abbreviations: ph.: phoneme, t.: tongue, vel./glot. apert.: velar/glottal aperture, compl. constr.: complex constriction. The consonants are listed using the conventions of the Speech Assessment Methods Phonetic Alphabet (SAMPA; Wells, 1997). “1” indicates the presence of the articulatory feature in the articulation of the phoneme; an empty field indicates its absence.

ph.	lips	t. tip	t. back	vel. apert.	glot. apert.	compl. constr.
p	1				1	
b	1					
t		1			1	
d		1				
k		1			1	
g		1				
N			1	1		
m	1			1		
n		1		1		
l		1				1
R			1			1
r		1				1
f	1				1	1
v	1					1
s		1			1	1
z		1				1
S		1			1	1
Z		1				1
j			1			
x			1		1	1
h						1

Execution of articulatory gestures in the stream of speech does not start from an invariant, zero position of articulators in the vocal tract. Rather, it depends on the immediate phonological context. A new articulatory gesture can require different movements for the production of one and the same phoneme, depending on its antecedent (the so-called “co-articulation effect” (Liberman, Delattre, and Cooper (1952)). Several lines of evidence from psycholinguistic research (summarized in Ziegler and Aichert (2015)) suggest that such variability in speech is possible owing to the phonological planning of articulatory gestures in hierarchical, non-linear constellations, which Ziegler and Aichert (2015) assume as the basis of their EoA model. This model adheres to the following other

principles. It (1) accommodates two kinds of tongue gestures: those of the tongue tip and those of the tongue back, (2) treats all vowels and diphthongs as involving exactly one gesture of the vocal tract, (3) treats all consonants as involving one gesture of either the lips, the tongue tip, or the tongue body plus a possible additional glottal aperture in voiceless phonemes or a velar aperture in nasal phonemes. Finally, it (4) treats fricatives and lateral and rhotic sounds as "complex" and plosives, nasals and vowels as "simple" constriction types (the last column of Suppl. Tab. 1).

Ziegler and Aichert's model (2015) assumes a probability p of correct articulation for all gestures of the vocal tract. It uses a number of weighting coefficients to account for the characteristics of the individual gestures as well as for gestural embedding (described below). Glottal apertures are weighted with the coefficient c_{glot} and velar apertures with the coefficient c_{vel}. When involved in the production of complex-constriction-type phonemes, the primary articulators, namely, the lips, the tongue tip, and the tongue back, are weighted with the coefficient c_{cnstr}. For the phoneme /S/, e.g., the gesture of the tongue tip is weighted with its occurrence in a fricative (c_{cnstr}), and the glottal aperture is weighted with c_{glot}. The probability of correct articulation of a combination of n gestures is obtained by multiplying the weighted probabilities p_1, p_2, ...p_n for each gesture. The probability $p_{/S/}$ of correct articulation of /S/ is therefore:

$$p_{/S/} = \underbrace{(p_1 \times c_{cnstr})}_{\text{tongue-tip gesture}} \times \underbrace{(p2 \times \ c_{glot})}_{\text{glottal aperture}}$$

Ziegler and Aichert's (2015) model specifies the relations between vocal-tract gestures on three higher levels of gestural embedding: (A) the level of consonant clusters, (B) the level of syllable structure, and (C) the level of prosody (Suppl. Fig. 2).

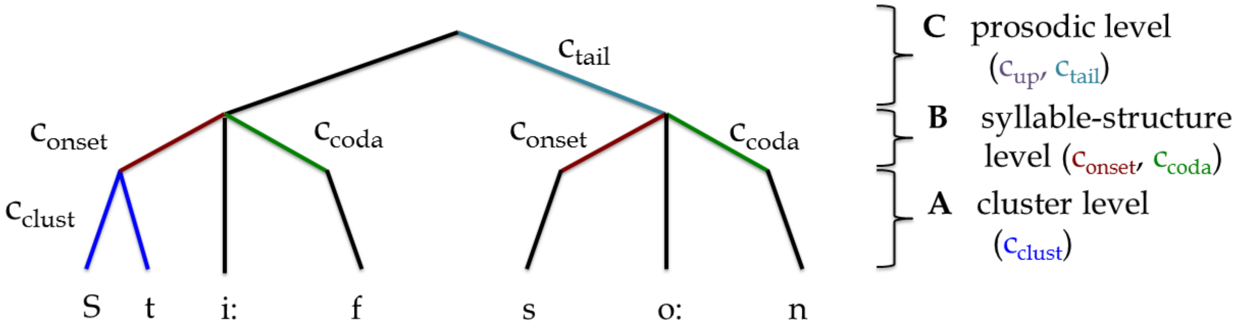

Supplementary Figure 2. A schematic illustration of the hierarchical relations between vocal-tract gestures relevant to the calculation of the ease of articulation index (EoA) on three levels of gestural embedding in Ziegler and Aichert's model (2015), illustrated on the example of the word "Stiefsohn" (Engl. "stepson"). **(A)**, Articulatory gestures that are part of a consonant cluster are weighted with c_{clust} on the cluster level. **(B)** They are weighted on the level of syllable structure for the occurrence in the onset or coda position within the syllable. **(C)** On the prosodic level, articulatory gestures are weighted with the coefficients c_{up} or c_{tail}, depending on their accentual-syllabic meter. See Methods and Suppl. Tab. 2 for definitions of these coefficients. Colored bars of the tree structure indicate that the respective coefficient needs to be applied in the EoA calculation of "Stiefsohn".

(A) Articulatory gestures that occur in a consonant cluster are additionally weighted with c_{clust}. Note that the model by Ziegler and Aichert (2015) accounts for the fact that articulation of a phoneme depends on its phonological neighborhood: If two adjacent phonemes involve the same primary articulatory organ (columns 2-4 of Suppl. Tab. 1) and have the same constriction

complexity (last column of Suppl. Tab. 1), the model counts the shared articulatory gesture not twice but once (a half for each phoneme). The likelihood of accurate articulation for /St/ in the word ‘Stiefsohn’ (Engl. ‘stepson’) is thus weighted as follows:

$$p_{/\mathrm{St}/} = \underbrace{\underbrace{(p_1 \times c_{cnstr} \times c_{clust})}_{\text{tongue-tip gesture}} \times \underbrace{(p_2 \times c_{glot} \times c_{clust} \times 0.5)}_{\text{glottal aperture}}}_{/\mathrm{S}/} \times \underbrace{\underbrace{(p_3 \times c_{glot} \times c_{clust} \times 0.5)}_{\text{glottal aperture}} \times \underbrace{(p_4 \times c_{clust})}_{\text{tongue-tip gesture}}}_{/\mathrm{t}/}$$

The gesture for devoicing, the glottal aperture gesture, is shared between /S/ and /t/, and it therefore multiplied by 0.5 for each phoneme. The tongue-tip gestures of /S/ and /t/ are not shared, since the type of constriction complexity changes from /S/ (complex) to /t/ (simple). The vocal-tract gestures that are not part of a consonant cluster do not receive additional weights on this level. On the level of syllable structure (B), all articulatory gestures in the syllable onset position, i.e., before the nucleus of the syllable, are weighted with c_{onset}. The probability of correct articulation of the phoneme /S/ in the word “Stiefsohn” is therefore:

$$p_{/S/} = \underbrace{(p_1 \times c_{cnstr} \times c_{clust} \times c_{onset})}_{\text{tongue-tip gesture}} \times \underbrace{(p_2 \times c_{glot} \times c_{clust} \times c_{onset} \times 0.5)}_{\text{glottal aperture}}$$

All gestures that occur in the coda position of a syllable are weighted with c_{coda}. The nucleus does not receive an additional weight on the level of syllable structure. On the prosodic level (C), the weighting of articulatory gestures depends on the word’s accentual-syllabic meter. The gestures that occur in the stressed syllable of an iambic, trochaic or amphibrach foot are never weighted. Articulatory gestures that appear in the weak syllable of a trochaic foot are weighted with c_{tail}, and gestures in the weak syllable of an iambic foot are weighted with c_{up}. In an amphibrach foot, the first syllable is analyzed as the upbeat syllable and the last syllable as the tail syllable with the respective c_{up} and c_{tail} coefficients. Once the contributions of each articulatory gesture within a word have been accounted for on all three levels of linguistic abstraction (Suppl. Fig. 2), the weighted probabilities are multiplied to calculate the probability of correct articulation for the entire word.

The weighting coefficients used to specify the relationships described above are provided in Suppl. Tab. 2, reproduced with permission from Ziegler and Aichert (2015). These authors obtained the coefficients using a model trained to predict the probability of correct articulation in AoS patients and validated them in a different group of 40 AoS patients based on a set of 48 words and 48 non-words; The model yielded a good estimate for accurate articulations, reaching an $R^2_{adj.}$ of 0.67 in the cross-validation trial. The suitability of their model and of these coefficients to describe the EoA of words is thus plausible.

Supplementary Table 2: Coefficient estimates for the full regression model, adapted with permission from Ziegler and Aichert (2015).

coefficient	estimate	standard error	significance level[a]
**p**	0.970	0.019	ns
c_{onset}	0.859	0.039	0.01
c_{coda}	0.896	0.036	0.05
c_{glot}	1.191	0.051	0.01
c_{vel}	1.188	0.058	0.01
c_{clust}	0.984	0.021	ns
c_{cnstr}	0.902	0.026	0.01
c_{up}	0.916	0.018	0.01
c_{tail}	1.010	0.017	ns

[a] 0.05/0.01: 95%/99% confidence interval (CI) excludes "1"; ns: 95% CI includes "1".

1.4. Application of the EoA model on our data

To apply the EoA model by Ziegler and Aichert (2015) on our data, we transcribed all content words selected from the patients' speech production based on the conventions of the Speech Assessment Methods Phonetic Alphabet (SAMPA; Wells, 1997).The advantages of this method of phonemic transcription are that it: is suited for the principal languages of the European Union, offers an alphabet that can easily be read by computer languages, and allows for easy transmission of the characters into the IPA alphabet. The transcription was carried out based on the patients' actual articulation. That is, if the patient produced the standard German word 'haben' (Eng. 'have' in the 3rd person plural) as /ham/, the phonological form used for further analysis was /ham/. This was done to ensure that the calculated EoA would reflect the motor planning requirements as closely to the actual speech as possible. We obtained SAMPA transcriptions for words produced in standard German using a CELEX[1]-based computer program by Dr. Aichert and colleagues. This program, designed for generation and analysis of language material depending on its compositional structure and frequency features, was kindly made available to us by the respective lab. Whenever automated transcription was not possible, i.e., in cases when the patients' articulation deviated from standard German pronunciation or when a word was not registered in CELEX, we transcribed the words manually in accordance with the SAMPA conventions. Next, we conducted automated tagging of the words' syllabic structure (i.e., which of the phonemes in the word were vowels and which were consonants) using the aforementioned software, manually annotated the words that were not registered in CELEX, and discarded all words that did not match the criteria of the EoA model.

The model by Ziegler and Aichert (2015) accounts for words with a certain accentual-syllabic meter. It is applicable on words whose prosodic contour falls into the categories stressed-unstressed, unstressed-stressed, or unstressed-stressed-unstressed. Note that the category of "stressed" syllables refers to the primary stress only. Words longer than three syllables

[1] CELEX: a lexical database that includes several corpora of German (Baayen et al., 1995)

therefore did not match these patterns and had to be excluded from the analysis. Some three-syllabic words also had to be discarded for the same reason. These were either words in which the first syllable was stressed, e.g., 'vorstellen' *(/fo:r-StEl-l@n/*, stressed-unstressed-unstressed; Engl. 'imagine') or in which the last syllable was stressed, e.g., 'optimal' (*/Op-ti:-ma:l/*, unstressed-unstressed-stressed; Engl. 'optimal'). The words discarded due to their deviant metric structure were: 6%(±2%) ADV, 24%(±6%) NN, and 10%(±3%) FV in all subjects on average (mean), corresponding to 10%(±3%) out of the total number of words (Suppl. Tab. 3) regardless of their PoS category. As one can see, the exclusion of words due to their prosodic contour did not result in losing much data in ADV and FV categories, while NN lost about ¼ of the words. This is because NN in German tend to have a larger number of syllables than the other investigated PoS, and also because NN have more composita. Representative examples of the latter in our data are 'Krankenhaus' (Engl. 'hospital', composed of 'krank' (Engl. 'sick') and 'Haus' (Engl. 'house')) and 'Hirnregion' (Engl. 'brain region', composed of 'Hirn' (Engl. 'brain') and 'Region' (Engl. 'region')).

Based on the annotations of the consonant cluster structure, syllabic composition, and the prosodic properties of the words, we calculated the EoA as described above with the help of a custom-made MATLAB-based program.

1.5. Automated correction of word, clause and speech epoch boundaries in the neurolinguistic data

This correction was performed to align word and speech starts to clause starts as well as word and speech ends to clause ends whenever the respective tag combinations occurred within the same time window for automated correction. The time window for automated correction was systematically varied in steps of 5 ms between 5 and 50 ms to the left and to the right from the tag. The software identified missing tags of the respective pair (i.e., word start/end, clause start/end, speech start/end) within the time window of interest. It also produced warnings, whenever impossible or unlikely durations which would likely need tag correction could be identified (e.g., negative or excessively long words, clauses or speech production epochs). To select an optimal time correction threshold for each subject, the outputs of automated correction at each threshold (i.e., the time window for automated correction) were checked by validating the plausibility of the resulting overlaps between the tags based on the content of the transcriptions. It was possible to tell from this comparison, whether a word start/end and a speech start/end in the respective clause were supposed to overlap or not. For instance, if an adverb start had the same timing as "cs" after the automated time correction, we checked if that adverb indeed corresponded to the clause-initial word in the transcription. The best correction thresholds ranged between 10 and 40 ms, depending on the subject. In rare cases, the best thresholds failed to account for 1-2 individual tags per subject. The timing of these tags was then manually adjusted upon automated correction at the best threshold selected.

2. Supplementary results

2.1. Composition of the selected linguistic material

More than a half of the gathered nouns could be used for the analysis, while the categories of full verbs and adverbs lost many words in comparison. This is because the latter two word categories showed considerably more repetitions within the lexical samples of the individual subjects. The reproducible proportional relations between and within the PoS categories analysed appear to reflect the subject-unspecific linguistic composition of spontaneously spoken German. The proportional relation between the total numbers of the gathered content words relative their numbers within the respective PoS category was reproducible in all subjects (Suppl. Tab. 3).

Supplementary Table 3: The amount of the collected and analysed linguistic material. Abbreviations: ling.: linguistic, ADV: adverbs, FV: full verbs, NN: normal nouns, №. w.: number of all words together regardless of their PoS category, №. cl.: number of clauses; sel./total: number of the respective language units selected for the analysis, divided by the number of such units gathered per subject in total, abs.: in absolute values, %: expressed in per cent rounded to the last integer; mean: the average percentage of the language units selected relative to the total number of these language units, the absolute maximum deviations from this value within our sample of subjects (S1-S5) are given in brackets.

s. / ling. unit	S1		S2		S3		S4		S5		mean
	**proportional relation sel./total**										
	**%**	**abs.**	**%**	**abs.**	**%**	**abs.**	**%**	**abs.**	**%**	**abs.**	**%**
**№. ADV**	29%	83/286	26%	65/248	28%	68/240	29%	156/535	32%	135/425	29% (±3%)
**№. FV**	37%	143/389	38%	115/302	40%	132/327	35%	260/741	34%	173/509	37% (±3%)
**№. NN**	64%	81/127	60%	60/100	51%	61/120	56%	177/317	52%	84/161	57% (±7%)
**№. w.**	38%	307/802	37%	240/650	38%	261/687	37%	593/1593	36%	392/1095	37% (±1%)
**№. cl.**	57%	218/383	57%	170/299	59%	190/325	60%	433/723	56%	282/506	58% (±2%)

2.2. Statistical testing of neurocorrelation results before and after residualization

In neurocorrelation analyses, we applied a sequence of tests which were either more or less conservative. They ranged from Bonferroni-corrected testing to uncorrected testing at different thresholds up to 5E-06. Note that even the least conservative test was thus relatively strict and it yielded spatially meaningful, focal neural effects. For each parameter, however, we sought to find out, which effects would survive the most conservative testing possible (i.e., the most conservative test and threshold at which any effects for the given parameter could be observed at all, highlighted in green in Suppl. Tab. 4). If Bonferroni-corrected testing yielded significant results, the result of this test was reported and visualized in the figures depicting neurocorrelation results (Figs. 6-7 in the main text), even though uncorrected testing at very conservative thresholds sometimes elicited very similar results (e.g., CVR in S3 in Suppl. Tab. 4). In total, effects for FRQ could be observed in all 5 subjects analysed, for NoS in 4 subjects, and CVR and EoA yielded significant effects in only 3 subjects. In terms of the numbers of electrodes with significant effects per subject per parameter,

spatially specific results could be observed: the numbers of electrodes with significant effects, identified as described above, ranging from 1 to 6 electrodes. The effects of threshold and test choice in neurocorrelations of the RSM with the psycholinguistic parameters are shown in Suppl. Tab. 4.

Supplementary Table 4: An overview of the outcomes when testing correlations between RSM responses and the linguistic parameters before residualization with the help of different statistical procedures. The outcomes of Bonferroni correction for the number of time-frequency bins and electrodes (Bonf.) and of uncorrected testing (unc.) at different statistical thresholds (thr.) are summarized for parameters (par.) FRQ (lemma frequency extracted from the linguistic corpus FOLK), EoA (ease-of-articulation index, Ziegler and Aichert 2015), NoS (number of syllables in a spoken word), CVR (consonant-to-vowel-ratio, i.e., the number of consonants in a spoken word divided by the number of vowels). “yes”: a test at the respective threshold elicited significant results for at least one electrode, the number of electrodes with significant effects/the total number of tested electrodes are given in brackets. The positive outcomes of the most conservative statistical test (Bonferroni correction at q<0.05 or uncorrected testing at the most conservative threshold per parameter per subject in the absence of Bonferroni-corrected effects) are highlighted by a green background. “no”: a test at the respective threshold elicited no significant results.

s.	test	thr.	par.			
			**CVR**	**EoA**	**FRQ**	**NoS**
**S1**	Bonf.	0.05	no	no	yes (1/56)	no
	unc.	5E-06	no	yes (3/56)	yes (4/56)	yes (2/56)
	unc.	1E-06	no	no	yes (1/56)	no
	unc.	5E-07	no	no	yes (1/56)	no
	unc.	1E-07	no	no	no	no
**S2**	Bonf.	0.05	yes (4/59)	yes (1/59)	no	no
	unc.	5E-06	yes (13/59)	yes (12/59)	yes (6/59)	yes (2/59)
	unc.	1E-06	yes (8/59)	yes (3/59)	no	yes (1/59)
	unc.	5E-07	yes (7/59)	yes (1/59)	no	yes (1/59)
	unc.	1E-07	yes (4/59)	no	no	no
	unc.	5E-08	yes (3/59)	no	no	no
	unc.	1E-08	no	no	no	no
**S3**	Bonf.	0.05	yes (1/40)	no	no	yes (1/40)
	unc.	5E-06	yes (5/40)	no	yes (6/40)	yes (2/40)
	unc.	1E-06	yes (1/40)	no	yes (3/40)	yes (1/40)
	unc.	5E-07	yes (1/40)	no	no	yes (1/40)
	unc.	1E-07	no	no	no	yes (1/40)
	unc.	5E-08	no	no	no	yes (1/40)
	unc.	1E-08	no	no	no	no
**S4**	Bonf.	0.05	no	no	no	no
	unc.	5E-06	no	yes (1/55)	yes (16/55)	yes (4/55)
	unc.	1E-06	no	no	yes (4/55)	yes (3/55)
	unc.	5E-07	no	no	yes (1/55)	yes (1/55)
	unc.	1E-07	no	no	no	no
**S5**	Bonf.	0.05	no	no	no	no
	unc.	5E-06	yes (2/62)	no	yes (1/62)	no
	unc.	1E-06	no	no	no	no

Suppl. Tab. 5 provides a neuroanatomical description of the effects observed prior to residualization for each parameter using the most conservative test and threshold. It specifies the range of frequencies within which the effects occurred, provides the MNI coordinates of the electrodes and gives functional descriptions of these electrodes obtained with the help of ESM.

Supplementary Table 5: Correlations between RSM responses and the linguistic parameters with the most conservative statistical test and threshold before residualization. Abbreviations: ele.: electrode name; frq. (Hz): frequency of the significant effect in Hz, frq. r.: frequency range of the effect, β: beta (15-30 Hz), Lγ: low gamma (35-45 Hz). Hγ: high gamma (50-150 Hz), the effects in gamma frequencies are in black font (visualized in Fig. 6 in the main text), the effects in lower frequencies are in grey font (not visualized); corr. pref.: correlation prefix, neg.: negative, pos.: positive; time rel. to w.: time of the effect relative to word production, bef.: before, dur.: during, aft.: after; MNI (x/y/z): coordinates of the electrode with a significant effect; str. area: structural area of the effect; funct. area (mon. ESM)/(bip. ESM): functional area identified with either monopolar or bipolar ESM; n./s.: electrode was not stimulated, n./.e.: electrode was stimulated but elicited no observable effect, ch.: chin, f.: finger, he.: head, l.: lip, t.: tongue, mot.: a motor response (a movement of the corresponding body part), sens.: a sensory response (a tactile sensation), speech: ESM-identified language-essential cortical site. °: a cortical site outside of the ESM-identified potentially speech-relevant cortex (Fig. 3A in the main text) which lay in its immediate neighbourhood; ^: a cortical site outside of the ESM-identified potentially speech-relevant cortex without ESM-identified potentially speech-relevant cortical sites in its immediate neighbourhood; overl. eff.: a significant effect of at least one other parameter occurred in the same time-frequency range (see Methods for a definition) when using the same statistical test and threshold. EMG: average relative spectral magnitude for electromyographic activity during word production at the subjects' left cheeks, DELL: EMG from the subject's left deltoid muscle, int.: intensity of the acoustic signal, ss_ws: temporal duration from speech start to word start in ms, ws_we: temporal duration from word start to word end in ms, we_se: temporal duration from word end to speech end in ms, abbreviation for the other parameters are the same as in Suppl. Tab. 4; "eff. surv. in postresid" indicates whether ("yes") or not ("no") the effect survived when repeating the same analysis after residualization (Suppl. Tab. 6), i.e., by correlating the RSM values with the residuals of the linear model predicting the parameter by all other parameters with which it had significant correlations; "=thr.": effect upon residualization occurred at the same electrode and in the same time-frequency range when using the same test and statistical threshold (Fig. 7 in the main text), "↑thr.": effect upon residualization occurred at the same electrode and in the same time-frequency range when using the same test at a more conservative threshold, **: a post-residualization effect occurred at the same electrode but in a different frequency range.

s.	par.	test	thr.	ele.	frq. (Hz)	frq. r.	corr. pref.	time rel. to w.	MNI (x/y/z)	str. area	funct. area (mon. ESM)	funct. area (bip. ESM)	overl. eff.	eff. surv. in postresid.
**S1**	**EoA**	unc.	5E-06	C1	40-	Lγ	neg.	bef.	-62/-55/27	IP	n./s.	speech	#	no
				D5	45	Lγ	neg.	dur.	-60/-15/44	SI	l. mot.	l. & he. mot.	NoS	no
				D6	75	Hγ	neg.	dur.	-58/-4/44	PM	l. & t. mot.	l. & he. mot.	#	no
	**FRQ**	Bonf.	0.05	E5	120-	Hγ	neg.	dur.	-54/-16/53	SI	thumb mot.	l. & he. mot.	#	yes (=thr.)
	**NoS**	unc.	5E-06	A5	45	Lγ	pos.	bef.	-66/-16/8	TC	n./s.	speech	no	no
				D5	45	Lγ	pos.	dur.	-60/-15/44	SI	l. mot.	l. & he. mot.	EoA	no
**S2**	**CVR**	Bonf.	0.05	B2	140	Hγ	pos.	bef.	-51/10/43	BR	n./e.	n./e.°	#	yes (=thr.)
				B3	135	Hγ	pos.	aft.	-51/-1/53	PM	ch. & t. mot.	ch. mot.	#	yes (=thr.)
				E4	125	Hγ	pos.	bef.	-67/-17/26	OP	n./e.	t. mot.	#	yes (=thr.)
				E5	115	Hγ	pos.	dur.	-66/-30/29	IP	n./e.	hand sens.°	#	yes (=thr.)
	**EoA**	Bonf.	0.05	C4	50	Hγ	neg.	aft.	-58/-14/47	SI	f. sens.	f. sens.^	#	no
	**FRQ**	unc.	5E-06	C3	40,	Lγ,	neg.	bef.	-58/-3/44	PM	ch. & l. mot.	hand sens.	no	no
				C7	30	β	neg.	bef.	-53/-44/54	IP	n./e.	thigh mot.^	#	no**
				E1	30	β	neg.	bef.	-63/13/15	BR	n./e.	t. mot.	#	no
				E5	40	Lγ	neg.	bef.	-66/-30/29	IP	n./e.	hand sens.°	no	no
				E7	50	Hγ	neg.	aft.	-63/-47/34	IP	n./e.	thigh mot.°	#	no
				H2	75	Hγ	neg.	dur.	-63/-6/-9	TC	n./s.	speech	#	no
	**NoS**	unc.	5E-07	B3	130	Hγ	pos.	bef.	-51/-1/53	PM	ch. & t. mot.	ch. mot.	ss_ws	no
**S3**	**CVR**	Bonf.	0.05	E2	145	Hγ	pos.	aft.	-65/-5/23	CS	n./s.	n./e.	EMG,	no
	**FRQ**	unc.	1E-06	F1	90	Hγ	pos.	aft.	-64/9/12	BR	n./s.	l. & t. mot.	no	no
	**NoS**	Bonf.	0.05	D7	75	Hγ	neg.	bef.	-58/-63/18	IP	n./s.	n./e.°	#	no
**S4**	**EoA**	unc.	5E-06	E8	40	Lγ	pos.	aft.	-56/-36/51	IP	f. sens.	f. sens.	EMG	no
	**FRQ**	unc.	5E-07	G8	130	Hγ	neg.	bef.	-62/-44/38	IP	n./s.	n./e.	EMG	no
	**NoS**	unc.	5E-07	E6	65	Hγ	pos.	bef.	-58/-18/46	SI	l. mot. &	l. & t. mot.	int.,	no
**S5**	**CVR**	unc.	5E-06	D8	25	β	neg.	bef.	-44/21/42	PF	n./s.	aura		yes (↑thr.)
				D8	75	Hγ	pos.	aft.	-44/21/42				DELL	yes (↑thr.)
				F6	120	Hγ	neg.	bef.	-39/-4/63	PM	hand mot.	hand mot.^	#	no
	**FRQ**	unc.	5E-06	B5	40	Lγ	pos.	dur.	-63/-5/24	SI	t. mot.	t. mot.	no	no

Supplementary Table 6: An overview of the outcomes when testing correlations between RSM responses and the linguistic parameters after residualization using different statistical procedures. All conventions as in Suppl. Tab. 4.

s.	test	thr.	par.			
			CVR	EoA	FRQ	NoS
S1	Bonf.	0.05	no	no	yes (1/56)	no
	unc.	5E-06	yes (1/56)	no	yes (2/56)	yes (1/56)
	unc.	1E-06	no	no	yes (1/56)	no
	unc.	5E-07	no	no	yes (1/56)	no
	unc.	1E-07	no	no	no	no
S2	Bonf.	0.05	yes (6/59)	no	no	no
	unc.	5E-06	yes (13/59)	no	yes (3/59)	no
	unc.	1E-06	yes (9/59)	no	no	no
	unc.	5E-07	yes (8/59)	no	no	no
	unc.	1E-07	yes (4/59)	no	no	no
	unc.	5E-08	yes (4/59)	no	no	no
	unc.	1E-08	yes (2/59)	no	no	no
	unc.	5E-09	yes (1/59)	no	no	no
	unc.	1E-09	yes (1/59)	no	no	no
	unc.	5E-10	yes (1/59)	no	no	no
	unc.	1E-10	no	no	no	no
S3	Bonf.	0.05	yes (1/40)	no	no	no
	unc.	5E-06	yes (4/40)	no	no	no
	unc.	1E-06	yes (2/40)	no	no	no
	unc.	5E-07	yes (1/40)	no	no	no
	unc.	1E-07	yes (1/40)	no	no	no
	unc.	5E-08	yes (1/40)	no	no	no
	unc.	1E-08	no	no	no	no
S4	Bonf.	0.05	no	no	no	no
	unc.	5E-06	yes (1/55)	no	yes (8/55)	no
	unc.	1E-06	no	no	yes (1/55)	no
	unc.	5E-07	no	no	no	no
S5	Bonf.	0.05	no	no	no	no
	unc.	5E-06	yes (2/62)	no	yes (2/62)	no
	unc.	1E-06	yes (1/62)	no	yes (1/62)	no
	unc.	5E-07	no	no	no	no

Suppl. Tab. 6 presents the results of the statistical testing after residualization. As can be seen from this table, EoA did not survive statistical testing with our specified tests and thresholds, NoS showed a significant effect in only one subject, FRQ yielded fewer effects but was still significant in three out of five subjects, and CVR showed significant results in all tested individuals. Suppl. Tab. 7 provides additional information about the locations of the neural effects observed together with their anatomical and functional descriptions.

In spite of certain spatial reproducibility, the effects observed before and after residualization in relation to the word complexity measures did not take place over broad ranges of gamma frequencies and over extended time periods (Suppl. Tabs. 5, 7), in contrast to the correlation effects observed with the linguistically-unspecific speech-duration-related parameters (Fig. 5 in the main text). There was little reproducibility with regard to the timing and to the exact frequential components of the spectrum, both within and between the linguistic parameters.

Supplementary Table 7: Correlations between RSM responses and the linguistic parameters investigated with the most conservative statistical test and threshold (after residualization). Abbreviations: α: alpha (5-10 Hz), eff. rel. to preresid: this column indicates whether the effect had been there already in the correlation analysis before residualization ("old") or if it was taking place at a different electrode and/or a different time-frequency range ("new"), **: an effect before residualization occurred at the same electrode but in a different frequency range; mid. f.: middle finger, aura: an ESM-induced feeling preceding an epileptic seizure, other conventions as in previous tables. The effects in gamma frequencies (in black font) are visualized for the linguistic parameters CVR and FRQ in Fig. 7 (main text).

s.	par.	test	thr.	ele.	frq. (Hz)	frq. r.	corr. pref.	time rel. to w.	MNI (x/y/z)	str. area	funct. area (mon. ESM)	funct. area (bip. ESM)	overl. eff.	eff. rel. to preresid.
**S1**	**CVR**	unc.	5E-06	H1	120	Hγ	pos.	bef.	-28/-55/71	SP	hand mot.	arm mot.^	no	new
	**FRQ**	Bonf.	0.05	E5	110	Hγ	neg.	dur.	-54/-16/53	SI	thumb mot.	he. & l. mot.	no	old (=thr.)
	**NoS**	unc.	5E-06	F3	70	Hγ	pos.	bef.	-49/-36/61	SI	little f. mot.	little & ring f. mot.°	no	new
**S2**	**CVR**	Bonf.	0.05	A8	150	Hγ	pos.	aft.	-29/-45/71	SI	n./e.	arm sens.-mot.^	no	new
				B2	140	Hγ	pos.	bef.	-51/10/43	PF	n./e.	n./e.	no	old (=thr.)
				B3	135	Hγ	pos.	aft.	-51/-1/53	PM	ch. & t. mot.	ch. mot.	no	old (=thr.)
				E4	125	Hγ	pos.	bef.	-67/-17/26	OP	n./e.	t. mot.	no	old (=thr.)
				E5	115	Hγ	pos.	dur.	-66/-30/29	IP	n./e.	hand sens.°	no	old (=thr.)
				F6	105	Hγ	pos.	aft.	-65/-41/22	TC	n./e.	speech	no	new
	**FRQ**	unc.	5E-06	C7	100	Hγ	neg.	aft.	-53/-44/54	IP	n./e.	thigh mot.	no	new**
				G3	145	Hγ	pos.	bef.	-68/-15/1	TC	n./s.	n./e.°	no	new
				H6	75	Hγ	pos.	dur.	-68/-44/-2	TC	n./s.	n./e.°	no	new
**S3**	**CVR**	Bonf.	0.05	D3	145	Hγ	pos.	aft.	-66/-16/28	OP	n./s.	n./e.^	no	new
**S4**	**CVR**	unc.	5E-06	F7	45	Lγ	neg.	bef.	-61/-32/43	IP	n./e.	n./e.°	EMG, int.	new
	**FRQ**	unc.	1E-06	B8	5-10	α	pos.	aft.	-30/-22/73	PM	index & mid. f. mot.	hand mot.^	EMG	new
**S5**	**CVR**	unc.	1E-06	D8	25	β	neg.	bef.	-44/21/42	PF	n./s.	aura°	no	old (↑thr.)
				D8	75	Hγ	pos.	aft.	-44/21/42	PF	n./s.	aura°	no	old (↑thr.)
	**FRQ**	unc.	1E-06	C6	35	Lγ	neg.	bef.	-62/5/34	PM	t. mot.	t. & l. mot.	no	new

2.3. Effects of residualization on the outcome of neurocorrelation analysis

Three scenarios could be observed as to how the neurocorrelation effects prior to and after residualization behaved in relation to each other (Suppl. Fig. 3):

(1) there was an effect prior to residualization which did not survive in post-residualization analysis;
(2) there was an effect prior to residualization which survived in post-residualization analysis at the same or at a less conservative statistical threshold;
(3) there was a new effect after residualization which was not there prior to it.

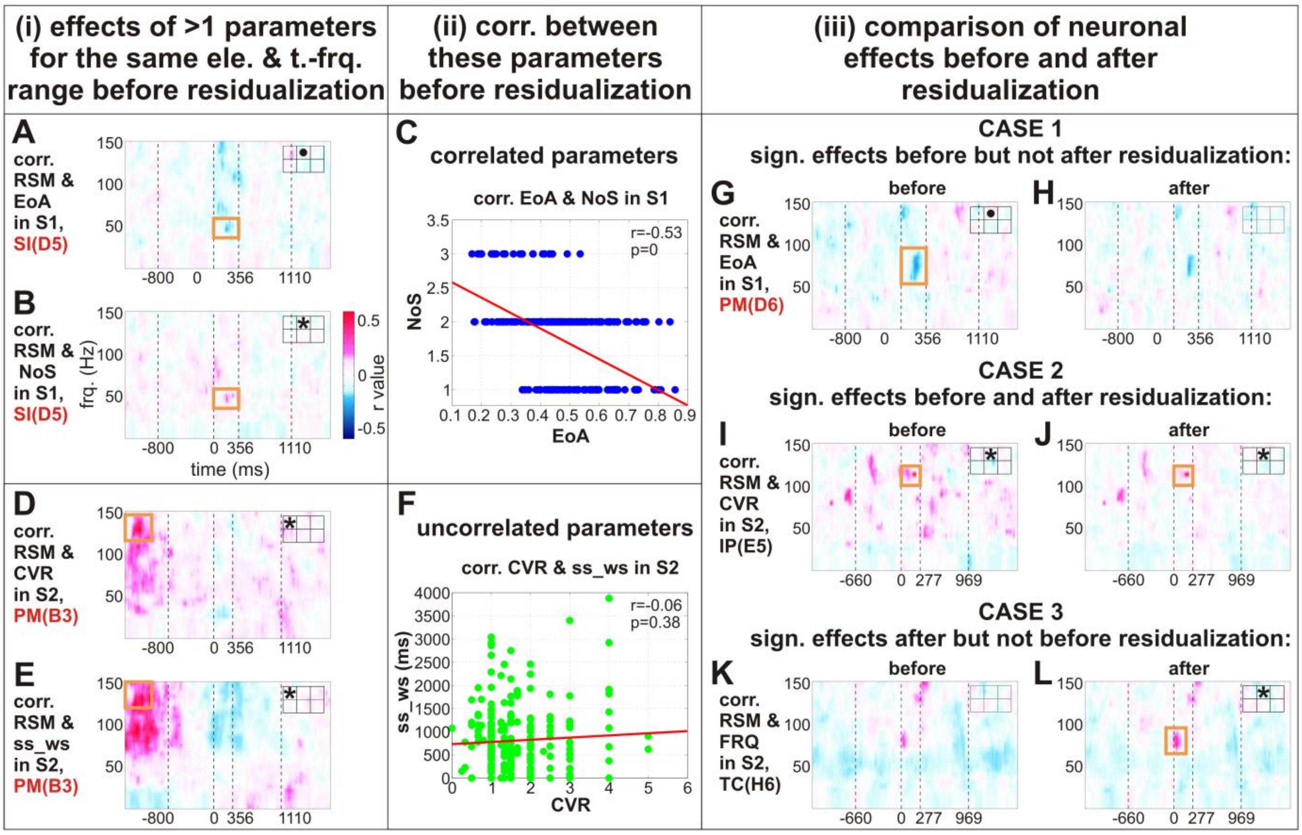

Supplementary Figure 3: Examples of RSM correlations with word-describing parameters (i) in relation to the collinearity structure between these parameters (ii) and (iii) a summary of the three impacts of removing multicollinearity in the linguistic data on the structure RSM correlations. Abbreviations and conventions: ele. & t.-frq. range: electrode and time-frequency range, >1 more than one, corr.: correlation, sign.: significant, orange frames circumscribe the time-frequency ranges in which the effects were significant at the displayed electrodes in the respective conditions, other abbreviations and conventions as in Figs. 5-7 in the main text. (1) shows examples of significant neural effects which occurred at the same mouth motor electrode (red font) and in the same time-frequency range (see Methods) with either different (**A, B**) or the same (**D, E**) correlation prefixes. These parameters either showed a significant correlation with each other (**C**) which could explain the different prefixes when correlating RSM values with these parameters (cf. A, B), or they were not significantly correlated (**F**) and thus could not explain the presence of significant RSM correlations with these parameters in the same time-frequency range at the same electrode (D, E, see Methods). A comparison of correlating RSM values with the linguistic parameters before residualization (**G, I, K**) with the outcomes of correlating the residuals (**H, J, L**) of the linear model predicting the respective parameter by all parameters with which it was significantly correlated in the respective subject resulted in changes post- compared to pre-residualization. These are listed and illustrated in (1) as cases 1-3.

The occurrence of these different options likely has several reasons. We assume that (1) may be due to the fact that mutually-correlated parameters bear meaningful information which cannot be disentangled into individual, parameter-specific components without its loss (Case 1 in Suppl. Fig. 3), (2) is likely an indication of the robustness of the effect and its relative independence on the presence of collinearity (Fig. 4 in the main text) in the linguistic data (Case 2 in Suppl. Fig. 3), and (3) can reflect the fact that residualization has removed some noise in the linguistic data, making hitherto concealed effects more clearly visible (Case 3 in Suppl. Fig. 3).

3. Supplementary discussion

3.1. Differences between neurocorrelation results between pre- and post-residualization data and the relation to previous work

CVR was the only parameter to show post-residualization effects in all subjects. This likely means that the areas of the pericentral cortex which showed effects related to CVR are sensitive to the distinction between vowels and consonants. The locations of these effects agree well with those reported using ECoG by Pei et al. (2011) using single-trial-decoding-based methods (cf. our Figs. 6A, 7A in the main text and their Fig. 5). Due to the lack of spatial or temporal reproducibility between subjects and/or between pre- vs. post-residualized effects in the other three complexity-relevant parameters, we shall refrain from functional interpretations of these effects. Since the present evidence is based on a relatively small sample of subjects, which is not uncommon in ECoG studies due to methodological reasons (Diekmann 2019), further work using larger samples and possibly automated procedures of data gathering will be required. In the research by Ziegler and Aichert (2015), the parameter EoA proved highly informative about errors in apraxia subjects. The present inconclusive results with regard to this parameter do not undermine this valuable previous work: while Ziegler and Aichert's (2015) evidence originated from lesion-based approaches in subjects with apraxia of speech, our data are correlative in their nature and they come from subjects with unimpaired language capacities. The former approach is capable of identifying mechanisms which involve not only individual areas on the cortical surface but likely reflect impairments in conduction of information between areas and also abnormal processes in the deeper cortical layers. Therefore, differences between our findings and those by Ziegler and Aichert (2015) are most likely attributable to methodological reasons.

The fact that lexical frequency, which has proven to modulate neural activity in some studies, did not yield reproducible post-residualization effects in the present work is possibly due to several reasons. First, previous neurolinguistic research dedicated to this phenomenon has largely been conducted in conditions of speech perception and elicitation, such as in lexical decision (Rugg 1990; Prabhakaran et al. 2006) or naming (van Petten and Kutas 1990; Graves et al. 2007) tasks, and it may be that word frequency effects in overt, spontaneous speech production have a distinctive neural infrastructure. Second, it is also conceivable that word frequency effects are not general in the sense that a negative correlation effect is equally visible regardless of the other word-describing parameters but that they are present only when some other parameters are accounted for. Kang (2012), who compared one- vs. two-syllable words in one ECoG-implanted subject, e.g., reported selective involvement of the middle temporal gyrus in the processing of two-syllabic words, and the inferior frontal gyrus proved active only when monosyllabic words were processed. In their EEG study, van Petten and Kutas (1990) observed interactions between word frequency and position of the word in a sentence. They showed that the amplitude of the N400 component of the event-related potential was larger for low-frequency words which occurred early in the presented sentences. These observations tie upon an ongoing discussion in (psycho-)linguistic research,

whether or not an effect of word frequency can be seen as an individual, robust phenomenon on its own, whether it is highly context-specific, or, alternatively, if such an effect is epiphenomenal to other linguistic factors. McDonald and Schillcock (2001) showed that contextual distinctiveness, or a corpus-derived measure of word probability in a given context, was a better predictor for lexical decision latencies than word frequency, and that word frequency effects could largely be explained by the presence of syntactic co-occurrence. A follow-up corpus-based investigation by Baayen (2010) addressed the extent to what other factors contributed to word frequency effects. This study, entitled “Demythologizing the word frequency effect,” showed that 90% of the variance in word frequencies could be predicted from other lexical properties and concluded that “frequency of occurrence, when understood in the sense of repeated experience, plays only a minor role in lexical processing[2]” (Baayen 2010: 437).

Our evaluation of the linguistic parameters is in agreement with Baayen’s observation that word frequency information can be correlated with other lexical properties and that it may hence be difficult to isolate a word frequency effect. Lemma frequency was strongly negatively correlated with NoS, weakly negatively correlated with CVR, and strongly negatively correlated with EoA. It also displayed a strong negative correlation with word duration (ws_we). A neurocorrelation analysis prior to residualization showed, as expected, predominantly negative correlations with activity in gamma frequencies (Fig. 6C), but these effects were largely gone when the analysis was performed after mutually-orthogonal parameters were extracted using a linear regression (Fig. 7B). The present findings also agree with those by Diekmann (2019), who did not find statistically robust effects of word frequency with the help of data from our corpus but using different methodological procedures. Note, however, that the absence of evidence (or, in our case, the presence of scarce evidence supporting the neural strength of a lexical frequency effect) should not necessarily be interpreted as evidence of absence (or, in our case, representational weakness): since previous neurolinguistic research showing word frequency effects has observed effects not only within but also beyond the cortical region covered in the FNLC, it is conceivable that recordings from other brain regions beyond our reach would still be informative. More ECoG research involving other brain regions may shed light on this question.

3.2. The range of spectral frequencies

Against our expectation of temporo-frequentially extended patterns, the correlation effects related to word complexity proved very local in time and frequency (e.g., Suppl. Fig. 3). At the same time, parameters describing the temporal duration from word start to speech start and also from word end to speech end (ss_ws and we_se) showed correlations with gamma activity in a broad range of frequencies and over more extended periods of time than the

[2] Since spontaneous language is inevitably associated with the problem of collinearity, lexical frequency is probably not the only linguistic property that can largely be explained by other contributors (Baayen 2010). With regard to the psychological plausibility of lexical properties, further research may be of interest which would permit accounting for a larger number of linguistic parameters and the extent to what they are influenced by other factors.

linguistic parameters investigated (Fig. 5 in the main text). This difference may suggest that mechanistic, linguistically-unspecific processes associated with executive functions during speech production are more dominantly represented in the pericentral cortex, compared with the parameters related to word complexity. These findings might also indicate that activity in the gamma range may contain subcomponents tuned to the distinctive linguistic features in temporally and frequentially narrow windows. Considering that the distribution of a frequency spectrum in the gamma frequencies depends on what particular cell types are active (Buzsáki, Anastassiou, and Koch 2012), it is also conceivable that these effects reflect the involvement of particular, localized cell groups which are tuned to individual higher-order processes. Since there is little evidence to support this possibility in current research (Gaona et al. 2011), further work will be needed to address this tentative speculation. Particularly studies with electrodes offering high spatial resolution of ECoG recordings (Wang et al. 2017) may be helpful to this end. The reader is likely wondering, whether the temporally and frequentially focalized correlation effects related to word complexity are attributable to the peculiarities of our strict statistical testing procedure, in which we reported only the effects which survived most conservative testing at most conservative thresholds (see Methods), and whether temporally and frequentially extended effects, as we were expecting, would have been observable using less conservative statistics. Unfortunately, this would generally not be the case: the effects related to our word complexity measures were always arranged in temporo-frequentially narrow clusters, as is shown on typical examples in Suppl. Fig. 3. Therefore, we believe that these properties are rather indications of moderate sensitivity of RSM values to parameters reflecting word complexity, compared to those related to the duration of the word-embedding speech production epochs.

3.3. Timing

We also find it difficult to draw conclusions with regard to the timing of the neural effects and its relation to (a) particular stage(s) of linguistic processing. This is especially the case with the parameters EoA and FRQ, which showed effects before, during, and after word production (Suppl. Tabs. 5, 7). If one assumes that the effects observed are not artefacts of statistical testing (see Methods), the disparate time points of activation observed might indicate involvement of these processes at multiple stages of word production and monitoring of the language output. In line with the assumption of syllabification taking place at relatively early stages of speech (Levelt and Meyers 2000), the effects related to NoS occurred either shortly before word onset or during word production. Due to the fact that this parameter lacked reproducibility with regard to the spatial and frequential properties, the related effects, too, need further validation and should be interpreted with caution. As concerning the parameter CVR, the timing of the effects appears interesting: CVR-related effects nearly always occurred either prior to or after but not during word production (with the exception of one electrode in the pre-residualized and one electrode in the post-residualized data, Suppl. Tabs. 5, 7). Our results do not allow drawing clear distinctions between anatomical areas with regard to the timing of the effects, since the same areas sometimes showed effects both before and after the word (e.g., the dorso-ventral prefrontal cortex converging on the premotor cortex or also

the parietal operculum). Network-structure analyses with functional connectivity measures (e.g., as in Kern et al. 2013) may be helpful to understand the relation between the time and region of CVR-related neural effects. Such an investigation was, however, beyond the scope of our study and further work in this direction may be of interest.

To sum up, this ECoG study reports on an innovative undertaking investigating word-complexity-related effects during non-experimental, real-world speech production by using a combination of linear regression and correlation approaches. We are aware of the fact that these linear methods may overlook non-linear relationships within the data. By removing linear trends, however, we made a step toward reducing mutual dependency on the individual parameters on each another, and this measure was sufficient to obtain parameter-specific neural effects (cf. Figs. 6 and 7 in the main text). In doing so, we were able to identify CVR as the linguistic parameter yielding most reproducible and robust positive correlations with gamma-range activity in the pericentral cortex. The lack of reproducibility in a number of characteristics of the neural signal underlying the other word-complexity-related parameters investigated may indicate their moderate representation in the neural signals studied. The fact that parameters related to speech duration, on the contrary, yielded strong, temporo-frequentially reproducible and spatially focalized effects in the articulatory motor cortex using exactly the same method (Fig. 5 in the main text) speaks for the feasibility of our approach and suggests that linguistically unspecific parameters associated with preparation for articulation and with articulation proper clearly dominate over the linguistically-relevant processes in the portion of the fronto-temporo-parietal region investigated.

References

1. Baayen, R. H. (2010). Demythologizing the word frequency effect: A discriminative learning perspective. *The Mental Lexicon, 5*(3), 436-461.
2. Baayen, R. H., Piepenbrock, R., & Gulikers, L. (1996). The CELEX lexical database (cd-rom).
3. Boersma, P., & Van Heuven, V. (2001). Speak and unSpeak with PRAAT. *Glot International*, *5*(9/10), 341-347.
4. Buzsáki, G., Anastassiou, C. A., & Koch, C. (2012). The origin of extracellular fields and currents—EEG, ECoG, LFP and spikes. *Nature reviews neuroscience*, *13*(6), 407.
5. Diekmann, B. C. (2019). *Wortproduktion im Satzkontext: Eine ECoG-Untersuchung*. [Doctoral dissertation, University of Freiburg], FreiDoc plus, Universitätsbibliothek Freiburg, doi: 10.6094/UNIFR/149963.
6. *Duden Online*. (no year). Online dictionary on www.duden.de (last visited on 11.04.2019).
7. Goldstein, L., & Fowler, C. A. (2003). Articulatory phonology: A phonology for public language use. *Phonetics and phonology in language comprehension and production: Differences and similarities*, 159-207.
8. Eisenberg, P. (2004). Der Satz: Grundriß der deutschen Grammatik, Bd. 2. Stuttgart: Verlag J. B. Metzler.
9. *FOLK*. (2012). Forschungs- und Lehrkorpus Gesprochenes Deutsch. Retrieved on March 25, 2016 from http://agd.ids-mannheim.de/folk.shtml (last visited on 11.04.2019).
10. Gaona, C. M., Sharma, M., Freudenburg, Z. V., Breshears, J. D., Bundy, D. T., Roland, J., ... & Leuthardt, E. C. (2011). Nonuniform high-gamma (60-500 Hz) power changes dissociate cognitive task and anatomy in human cortex. *Journal of Neuroscience*, *31*(6), 2091-2100.
11. Levelt, W. J., & Meyer, A. S. (2000). Word for word: Multiple lexical access in speech production. *European Journal of Cognitive Psychology*, *12*(4), 433-452.
12. Graves, W. W., Grabowski, T. J., Mehta, S., & Gordon, J. K. (2007). A neural signature of phonological access: distinguishing the effects of word frequency from familiarity and length in overt picture naming. *Journal of cognitive neuroscience*, *19*(4), 617-631.
13. Kang, S. (2012). *Neural basis of the word frequency effect and its relation to lexical processing*. [UC Berkeley Phonology Lab Annual Report (2012), http://linguistics.berkeley.edu/phonlab/documents/2012/Kang_word_freq.pdf (last visited on 11.04.2019)].
14. Kortmann, B. (2020). Syntactic variation in English: A global perspective. *The handbook of English linguistics*, 299-322.
15. Liberman, A M, P C Delattre, and F S Cooper. 1952. "The Role of Selected Stimulus Variables in the Perception of the Unvoiced Stop Consonants." *American Journal of Psychology* 65: 497–516.
16. McDonald, S. A., & Shillcock, R. C. (2001). Rethinking the word frequency effect: The neglected role of distributional information in lexical processing. *Language and Speech*, *44*(3), 295-322.
17. Musan, R. (2009). *Satzgliedanalyse.* Heidelberg: Universitätsverlag Winter.
18. *Netzverb (no year)* www.verbformen.de (last visited on 11.04.2019).
19. Ohala, J. J., Browman, C. P., & Goldstein, L. M. (1986). Towards an articulatory phonology. *Phonology*, *3*(1), 219-252.
20. Pei, X., Barbour, D. L., Leuthardt, E. C., & Schalk, G. (2011). Decoding vowels and consonants in spoken and imagined words using electrocorticographic signals in humans. *Journal of neural engineering*, *8*(4), 046028.

21. Prabhakaran, R., Blumstein, S. E., Myers, E. B., Hutchison, E., & Britton, B. (2006). An event-related fMRI investigation of phonological – lexical competition. *Neuropsychologia*, *44*(12), 2209-2221.
22. Rugg, M. D. (1990). Event-related brain potentials dissociate repetition effects of high-and low-frequency words. *Memory & Cognition*, *18*(4), 367-379.
23. Selting, M., Auer, P., Barth-Weingarten, D., Bergmann, J. R., Bergmann, P., Birkner, K., Couper-Kuhlen, E., Deppermann, A., Gilles, P., & Günthner, S. (2009). Gesprächsanalytisches Transkriptionssystem 2 (GAT 2). *Gesprächsforschung: Online-Zeitschrift Zur Verbalen Interaktion, 10*, 353-402.
24. Van Petten, C., & Kutas, M. (1990). Interactions between sentence context and word frequency in event-related brain potentials. *Memory & cognition*, *18*(4), 380-393.
25. Walker, M. B., & Trimboli, C. (1982). Smooth transitions in conversational interactions. *The Journal of Social Psychology*, *117*(2), 305-306.
26. Wang, X., Gkogkidis, C. A., Iljina, O., Fiederer, L. D., Henle, C., Mader, I., ... & Ball, T. (2017). Mapping the fine structure of cortical activity with different micro-ECoG electrode array geometries. *Journal of neural engineering*, *14*(5), 056004.
27. Wells, J. C. (1997). SAMPA computer readable phonetic alphabet. *Handbook of standards and resources for spoken language systems*, *4*, 684-732.
28. Ziegler, W., & Aichert, I. (2015). How much is a word? Predicting ease of articulation planning from apraxic speech error patterns. *Cortex*, *69*, 24-39.
29. Ziegler, W. (2008). Apraxia of speech. *Handbook of clinical neurology*, *88*, 269-285.
